# Culture and the Social Clock: Cultural Differences in the Optimal Timing of Life

**DOI:** 10.1177/01461672251362514

**Published:** 2025-09-01

**Authors:** Lu Zang, Heejung Kim

**Affiliations:** 1 University of California, Santa Barbara, USA

**Keywords:** culture, social clock, filial piety, self-conscious emotions

## Abstract

People typically hold personal views regarding the appropriate age ranges for significant life events, such as starting college, getting married, or having kids. Such socially prescribed timetables have been termed the social clock. In this paper, we investigate how and why the rigidity (or flexibility) of the social clock may vary across cultures. In three studies (two preregistered), participants from China and the United States were asked to provide the earliest and the latest ages they think appropriate for engaging in several life events. We operationalized the social clock’s rigidity as the width of the time windows for these life events. We found notable cultural differences: The social clock was more rigid in China than in the United States, and filial piety beliefs are likely explanations for these differences. We further assessed the anticipated negative self-conscious emotions associated with deviation from the social clocks. Societal implications and future directions were discussed.

The Master said: “At fifteen, I had my mind bent on learning.

At thirty, I stood firm.

At forty, I had no doubts.

At fifty, I know the decrees of Heaven.

At sixty, my ear was an obedient organ for the reception of truth.

At seventy, I could follow what my heart desired, without transgressing what was right.”

—from *The Analects of Confucius* (206 BC–220 AD)

Two thousand five hundred years ago, Confucius, at the sunset of his life, gathered his disciples and shared with them his timing of life, and his teachings still resonate among East Asians (Huang & Charter, 1996). Beyond the laudable goals, what is notable about this teaching is its emphasis on the prescribed age by which one should achieve the goals. Such emphasis is also reflected in current East Asian culture. Terms such as “leftover women”—women who remain unmarried in their late twenties and beyond—are commonly used in China (Spencer, 2016). Despite the changing demographics (e.g., decreasing birthrate) in Korea, Koreans perceive strong pressure to conform to social expectations about life milestones (Cho & LoCascio, 2018). This particular view on age-related expectations may contrast with the view prevalent in most Western cultures. In these cultures, age-based expectations about life milestones do not seem as strong. It may be deeply ingrained in people’s thinking in Western cultures that it is never too early or too late to pursue dreams, pick up new skills, and fall in love. For instance, it is not uncommon to encounter “nontraditional” students (e.g., undergraduate students older than age 25) enrolling in schools—cases that have become more prevalent in the West over the past decades (e.g., Casselman, 2013).

These sentiments illustrate that people hold divergent views in different cultures regarding not only the appropriate ages for significant life events, such as beginning college, getting a full-time job, getting married, or becoming a parent, but also the relative importance of age as a marker of success for these life milestones. In psychology, these age-related social expectations have been termed the *social clock* (Neugarten, 1976). However, although the term was introduced half a century ago, it has received relatively scant attention.

This relative absence of empirical attention to the topic of the social clock is perhaps due to the fact that over ninety percent of psychological research has been conducted in predominantly individualistic cultures (Dan, 2010), where age expectations tend to be more relaxed. We propose that the social clock may be an important concept that has significant implications for individual psychological well-being in more collectivistic nations such as China. Thus, the present research examines whether the social clock’s rigidity differs across cultures and, if so, what psychological factors explain the cultural difference. We do so by comparing individuals’ expectations of the social clock in two countries that represent individualistic and collectivistic cultures (i.e., the United States vs. China) and considering filial piety beliefs as an underlying reason for the difference. In addition, the current research examines how the social clock’s rigidity is linked to self-conscious emotions in these cultures, highlighting the psychological consequences of deviating from the social clock.

## The Social Clock

The social clock is a culturally defined timeline for life milestones, such as marriage, childbirth, and starting the first job (Neugarten, 1976). As one of the socially prescribed age norms, the social clock influences consecutive changes in behavior and self-perceptions across time in deciding whether people have made good progress in achieving life milestones (Helson & McCabe, 1994).

Previous studies showed that the social clock exists in many cultures (e.g., Lee & Payne, 2010; Byrd & Breuss, 1992; Gee, 1990; Peterson, 1996). For example, studies in New Zealand and Australia revealed that people show considerable agreement regarding the “correct” age at which people are expected to attain various life milestones (Byrd & Breuss, 1992; Peterson, 1996). Also, the social clock tends to vary among different types of life events. In a study conducted in two British Columbia cities (Gee, 1990), proportionately more women provided “right ages” for family events (e.g., getting married) than for nonfamily events.

Studies have also examined psychological factors affecting adherence to the social clock and the consequences of nonadherence to it. For example, women with higher scores on achievement via conformance (i.e., the desire to do well) and intellectual efficiency are more likely to conform to the feminine social clock (Helson et al., 1984). Studies conducted in the United States show that although being late in achieving desirable life events based on the statistical age norms is associated with increased psychological distress, such greater distress was not caused by a lack of social resources or negative social sanctions (Rook et al., 1989). In addition, being on time with life transitions does not invariably offer psychological advantages (Rook et al., 1989), the patterns of which may occur in more individualistic (compared to more collectivistic) cultures with a presumably more flexible social clock. However, it remains unknown how the social clock’s rigidity differs across cultures and how deviation from the social clock is associated with the anticipated emotional well-being across cultures.

## Role of Filial Piety

We reason that the cultural dimension of individualism–collectivism, in particular, the sense of social duty associated with this cultural dimension, leads to variations in the social clock’s rigidity. In the present research, we focused on two national cultures, the United States and China, which differ in the individualism–collectivism cultural dimension (Hofstede, 2011; Pelham et al., 2022).

China, with its long history of imperial rule, agrarian economy, and Confucian tradition, has historically placed a strong emphasis on group harmony and collective goals (e.g., Triandis, 1999). Confucianism, in particular, has deeply influenced Chinese society by promoting respect for authority, familial loyalty, and social order, fostering a collectivistic mindset that emphasizes collective goals (e.g., Triandis et al., 1988). These traditions continue to have a noticeable effect on Modern China. The United States, on the other hand, is a relatively newly settled country founded on principles of liberty, individual rights, and the Puritan idea of self-reliance. Rooted in its history of a “frontier spirit” (Kitayama et al., 2009), the United States emerged as a highly individualistic society where individual rights and freedoms are paramount.

Consequently, people from the two national cultures view interpersonal duties differently. Specifically, people from the United States are more inclined to prioritize self-expression, uniqueness, and personal choice, whereas people from China are more likely to place a stronger emphasis on social obligation and interpersonal responsibilities (e.g., Kim & Markus, 1999; Markus & Kitayama, 1991; Triandis et al., 1985). That is, in more collectivistic cultures, such as China, relative to more individualistic cultures, such as the United States, people tend to consider the desires and needs of those in their social group, adopt a more rigorous view of social responsibilities, and judge failure to assist another in moral terms (Cross et al., 2000; Miller et al., 1990).

While the cultural importance of social duties and responsibilities is found in many collectivistic cultural contexts, the manifestation of the concept varies across specific cultural regions (Campos & Kim, 2017). For example, the concept may take the form of familismo in Latin American cultures (e.g., Ayón et al., 2010) or filial piety in East Asian cultures (e.g., Ho, 1996). In the current study, as we compare the United States with China, we chose a social obligation—*filial piety*—that is especially important for the Chinese as a potential psychological factor explaining the cultural differences. Originating in ancient Chinese culture, filial piety is a virtue shared in Confucianism, Chinese Buddhism, and Daoist ethics. It highlights the beliefs and behaviors of fulfilling the duties of the younger generation in respecting and taking care of their parents and elders (e.g., Chan & Tan, 2008; Ho, 1996). In Chinese culture, which values filial piety, individuals are responsible for bringing honor to their families and carrying out their parents’ desires and dreams even after their deaths (Kohn, 2004; Yeh & Bedford, 2004).

For thousands of years, filial piety has been a crucial cultural attribute in China and its neighboring countries (Chan & Tan, 2008), and it continues to prevail even among Americans of Asian descent (Yeh & Bedford, 2003). Researchers have claimed that filial piety, communalism, and familism are “birds of a collectivist feather,” clustering into a single latent construct known as *family or relationship primacy*, a concept that emphasizes interdependence and interpersonal obligations and is central to Chinese culture (Schwartz, 2007; Schwartz et al., 2010).

In Chinese culture that cherishes the virtue of filial piety, life events such as getting married and having children are vital for continuing the family line and producing descendants as a defense against existential threats such as mortality (Qi, 2022). Besides, life events such as attending college and acquiring a full-time job can bring honor and secure resources for the family. As such, in China, the social clock is likely to be more rigid to better serve as a guideline for individuals to fulfill their social obligations. By contrast, in the United States, where independence and uniqueness are cherished (Kim & Markus, 1999; Markus & Kitayama, 1991), and filial piety, or more broadly, individuals’ sense of duties, is less commended, the timing of essential life milestones may have less to do with societal expectations. Therefore, we reason that cultural differences in the social clock’s rigidity are explained by corresponding cultural differences in filial piety.

## Social Clock and Emotional Well-being

Given that filial piety is a key manifestation of collectivism in Chinese culture, filial piety practice could matter for emotional well-being, as people who adhere closely to it are inclined to feel guilty and shameful if they go against the principles (see Bedford, 2004; Bedford & Hwang, 2003). Consequently, in China, those whose personal life timing deviates from the social clock may experience more *self-conscious emotions*, such as shame and guilt (e.g., Tracy et al., 2009), because failing to achieve life milestones indicates a failure to uphold filial piety. We focus on self-conscious emotions because deviation from the social clock may contribute to a discrepancy between one’s actual self and ought self, which can produce specifically anxiety-related emotions instead of broad negative affect (Higgins, 1987). Taken together, we reason that the link between fitting in the social clock and emotional well-being would be stronger in China than in the United States due to their differences in filial piety.

## The Present Research

The present research examines whether and how the social clock’s rigidity (or flexibility) differs across cultures. To our knowledge, no prior work has shed light on these inquiries. By examining the aspects of the social clock, our research presents a cultural perspective on an important yet often overlooked factor that influences individuals’ emotional well-being.

We measure the social clock’s rigidity using the width of “time windows” for specific life events. We ask participants to provide the earliest and latest ages they think are appropriate for accomplishing various life milestones. The time window for each life event is calculated by subtracting the earliest age from the latest age. The narrower (vs. wider) the time windows for life events, the more rigid (vs. flexible) the social clock is.

In three studies, we compared the time windows for several life events in the United States and China to examine whether and how their social clock’s rigidity differs. We also measured how deviation from the social clock, both from the upper bound (Studies 1a and 1b) and the lower bound (Study 2), impacts anticipated emotional well-being. Across all studies, we hypothesized that the social clock would be more rigid in China than in the United States. We also hypothesized that deviation from the social clock would be associated with more anticipated negative emotional outcomes in China than in the United States. Further, we explored the role that filial piety plays in these cultural differences.

Study 1a was exploratory. The hypotheses, analysis plans, and sampling method (and exclusion rules) for Study 1b were preregistered (https://aspredicted.org/7R8_GXN), as were those for Study 2 (https://doi.org/10.17605/OSF.IO/45BUW). Materials and de-identified data, along with their codebooks and data analysis scripts, are made publicly available at https://doi.org/10.17605/OSF.IO/QN23K. Additional measures and results are available in the supplementary materials.

## Study 1a

In Study 1a, we compared the variations in the widths of the time windows suitable for engaging in four life events (career or family related) between the United States and China to test the hypothesis that social clocks are more rigid in China than in the United States and to explore the role of filial piety beliefs in explaining such cultural differences. Moreover, we examined how deviation from the upper bound of the social clock would impact individuals’ emotional well-being across cultures. We hypothesized that deviation from the upper bound of the social clock would be more distressing for the Chinese than Americans.

### Participants

Informed by studies on comparable topics (e.g., Helson et al., 1984; Peterson, 1996), we set our sample size for Study 1 at 600 (300 per culture).^1^ We recruited 478 participants in the United States via the Prolific platform (*N* = 167) or an introductory psychology subject pool (*N* = 311), and 574 participants in China via WeChat, a Chinese social media. Participants who failed to complete the survey or declined to share anonymous data were excluded from the analyses. Participants who listed a lower value for the upper limit than for the lower limit were also excluded. Our final sample (*N* = 640) includes 343 American participants and 297 Chinese participants (Table 1).^2^

**Table 1.** Breakdown of Demographics in the United States and China in Study 1a.

Culture	Age *M* (*SD*)	Gender (%)	Ethnicity (%)	College degree (%)
The United States (*N* = 343)	21.73 (4.35)	Male: 37.9 Female: 58.9 Nonbinary/Other: 3.2	European American: 43.1 Hispanic/Latino: 28.6 Asian American/Pacific Islander: 12.8 Multiracial/Biracial: 9.3 Black/African American: 3.2 Other: 2.6 Native American/Alaskan: 0.3	24.2
China (*N* = 297)	24.20 (5.19)	Male: 43.4 Female: 56.2 Nonbinary/Other: 0.3	Han Chinese: 96.0 Ethnic Minorities: 4.0	94.3

### Measures

After consenting, participants completed an online survey with items following the subsequent order. The English version was translated into simplified Chinese for Chinese participants using the backtranslation method. The content of the Chinese version is identical to that of the English version, except for some demographic questions, given national differences in these measures (e.g., in education systems).

#### Time Window Blocks

##### Perceptions of the Social Clock

Participants were presented with six time window blocks. In each block, we first measured participants’ perceptions of the social clock by asking them to provide the earliest and the latest ages they thought appropriate for engaging in a particular event (Table 2). For example, participants read the description: “Assuming a person wants to attend a college. Please provide a range of ages that you think is appropriate for the person to start attending a college.” Then, participants were instructed to scroll two sliders (ranging from 10 to 90) to indicate the earliest (or lower bound) and the latest (or upper bound) ages they thought appropriate for the person to start attending college, respectively.

**Table 2.** Sequences of the Questions Measuring Time Windows in Study 1a.

Life Event category	Block 1	Block 2	Block 3	Block 4	Block 5	Block 6
Subcategory	Career-related life events		Family-related life events			
Specific life event	Starting college	Getting a full-time job	Getting married (male)	Getting married (female)	Having a first child (male)	Having a first child (female)

As family-related life events may be gender-dependent, we measured participants’ perceptions of the earliest and the latest ages appropriate for a male or a female to participate in each life event. The structure and phrasing of questions were similar to those for career-related life events. The time window for each life event is calculated by deducting the earliest age (or lower bound age) from the latest age (or upper bound age).

##### Anticipated Negative Emotional Outcomes of Deviation from the Social Clock (Upper bound)

We measured the anticipated negative self-conscious emotions resulting from deviation from the upper bound of the social clock. For instance, participants read: “Assuming you want to attend a college—Imagine you didn’t start attending college by the time you are reaching *this upper bound* of the appropriate age for attending college (i.e., by age [quoting the numeric value of the upper bound age they entered previously]). Please indicate how much you would feel the following emotions if you are not fulfilling the age-appropriate expectation.” Participants were presented with four emotional states, that is, “regretful,” “ashamed,” “anxious,” and “guilty,” and reported how much they would feel each emotion on a 5-point Likert-type scale (1 = *not at all* to 5 = *a great deal*). The structure and phrasing of questions were comparable in all blocks. As the reliabilities of ratings for the four anticipated negative emotions were high (*α*s > .85), we averaged them into a single composite score within each block.

#### Filial Piety Beliefs

The 12-item filial piety scale (Fu et al., 2020) was used to assess the extent to which one endorses filial piety beliefs. Example items include “Children should ask after their parents’ well-being” and “Children should try their best to honor their parents.” Participants reported their agreement or disagreement with each statement on a 5-point Likert-type scale (1 = *strongly disagree*, 5 = strongly agree). Item scores were averaged to create a composite score (α_US_ = .85; α_CH_ = .87). Higher scores indicated a higher endorsement of filial piety.

#### Additional Measures

The study included several measures to examine potential alternative psychological explanations. Analyses examining the roles of these additional factors are reported in the supplementary materials (pp. 4–5). In addition, the study included measures of the intersubjective perceptions of the social clock (i.e., participants’ perceptions of what a typical person in their country would consider appropriate for engaging in each life event) and anticipated negative emotional outcomes of deviation from the intersubjective social clock (i.e., the anticipated negative self-conscious emotions resulting from deviation from the upper bound of the intersubjective social clock). Their associated results will be outlined in a separate research paper (Zang & Kim, in preparation). Unless otherwise specified, the “social clock” in this paper refers to the subjective, not intersubjective, social clock. See the supplementary materials (pp. 1–2).

#### Demographic Covariates

Basic demographic information, including age, gender, education, and subjective socioeconomic status (SES; Adler et al., 2000), was measured as potential covariates.

### Results

#### Correlations between Variables

We first calculated the descriptive statistics of the variables and bivariate correlations between them in the United States and China (Table 3).

**Table 3.** Descriptive Statistics of the Variables and Bivariate Correlations in the United States and China in Study 1a.

	The United States (*N* = 343)								China (*N* = 297)							
Variable	*M* (*SD*)	1	2	3	4	5	6	7	*M* (*SD*)	1	2	3	4	5	6	7
1. Time window for career-related life events	39.36 (19.09)	—							17.69 (16.79)	—						
2. Time window for family-related life events	29.79 (14.16)	.60***	—						15.77 (12.44)	.69***	—					
3. Filial piety beliefs	3.13 (0.60)	−.24***	−.29***	–					3.66 (0.67)	−.42***	−.41***	—				
4. Anticipated negative emotional outcomes of deviation from the career-related social clock (upper bound)	3.13 (1.03)	−.15**	−.19***	.11*	—				3.07 (.99)	−.38***	−.28***	.21***	—			
5. Anticipated negative emotional outcomes of deviation from the family-related social clock (upper bound)	2.60 (1.06)	−.11*	−.17**	.26***	.52***	—			2.54 (1.04)	−.31***	−.31***	.46***	.58***	—		
6. Age	21.73 (4.35)	.14*	.13*	−.11*	−.22***	−.23***	—		24.20 (5.19)	−.03	−.01	−.01	−.11	.02	—	
7. SES	5.65 (1.78)	−.06	−.06	.24***	.12*	.19***	−.31***	—	5.64 (1.61)	−.01	.05	−.01	−.04	−.07	.10	—

*Note.* SES = socioeconomic status.

* *p* < .05. **p < .01. ***p < .001.

#### Cultural Differences in the Time Windows

**Figure 1.**
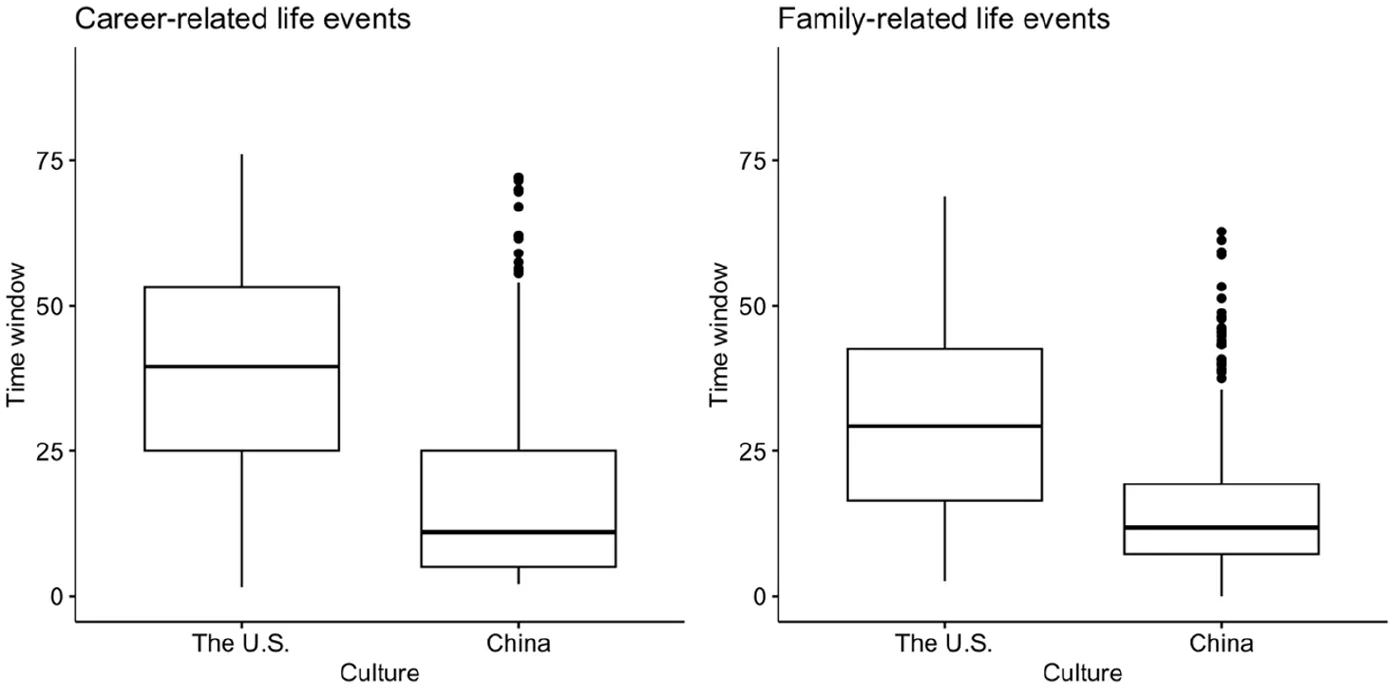
The widths of the time windows for career-related and family-related life events in the United States and China in Study 1a.

To test our key hypothesis, we examined whether culture affects the social clock’s rigidity by analyzing the time windows for career-related and family-related life events (Figure 1). In all analyses, we included age, gender, SES, and education as covariates.

We first examined the cultural differences in the time window for career-related life events. A generalized estimating equation (GEE) with robust standard errors (SEs) on the time window for career-related life events with culture (the United States vs. China) as a predictor with the covariates revealed a significant main effect of culture,^3^ Wald χ²(1, *N* = 640) = 182.33, *p* < .001. The time window for career-related life events was narrower in China (*M* = 16.02, robust *SE* = 1.11, 95% CI [13.84, 18.20]) than in the United States (*M* = 40.80, robust *SE* = 1.78, [38.49, 43.11]), *B* = 24.78, robust *SE* = 1.84, [21.18, 28.38].

We then ran the same analysis for the cultural differences in the time window for family-related life events. Similarly, we found a significant main effect of culture, Wald χ²(1, *N* = 640) = 117.94, *p* < .001. The time window for family-related life events was narrower in China (*M* = 15.03, robust *SE* = .84, 95% CI [13.39, 16.67]) than in the United States (*M* = 30.43, robust *SE* = .91, [28.65, 32.21]), *B* = 15.40, robust *SE* = 1.42, [12.62, 18.18]. When we examined lower and upper bound ages separately, we found significant cultural differences in most of the upper and lower bound ages, although the time windows are mainly driven by the differences in the upper bound ages (Table 4).^4^

**Table 4.** Descriptive Statistics and Cultural Differences in the Time Window for Each Life Event, as well as in the Lower and Upper Bound Age in Study 1a (Controlled for Demographic Covariates).

Specific life event	Culture	Lower bound age Mean (*SD*) Median	Wald χ²(1, *N* = 640) and B for the effect of culture on the lower bound age	Upper bound age Mean (*SD*) Median	Wald χ²(1, *N* = 640) and B for the effect of culture on the upper bound age	Time window Mean (*SD*) Median	Wald χ²(1, *N* = 640) and B for the effect of culture on the time window
Starting college	The United States	17.35 (2.91) 17.00	Wald χ² = .02, *B* = −0.05	60.26 (23.78) 60.00	Wald χ² = 143.77, *B* = 26.91***	42.91 (24.29) 42.00	Wald χ² = 137.67, *B* = 26.96***
	China	17.43 (2.16) 18.00		37.72 (21.89) 28.00		20.29 (22.36) 11.00	
Getting a full-time job	The United States	18.45 (2.41) 18.00	Wald χ² = 150.95, *B* = −3.58***	54.27 (20.91) 60.00	Wald χ² = 95.11, *B* = 19.02***	35.81 (21.42) 40.00	Wald χ² = 126.04, *B* = 22.60***
	China	21.74 (3.28) 22.00		36.82 (14.96) 30.00		15.08 (15.85) 9.00	
Getting married (male)	The United States	22.70 (4.02) 22.00	Wald χ² = 7.36, *B* = −1.92**	64.87 (21.65) 61.00	Wald χ² = 121.89, *B* = 22.26***	42.17 (22.85) 40.00	Wald χ² = 136.77, *B* = 24.19***
	China	24.10 (5.00) 24.00		43.33 (18.31) 36.00		19.23 (18.95) 12.00	
Getting married (female)	The United States	21.80 (3.43) 22.00	Wald χ² = 10.77, *B* = −1.18**	61.10 (22.84) 60.00	Wald χ² = 78.42, *B* = 18.85***	39.30 (23.48) 35.00	Wald χ² = 83.71, *B* = 20.03***
	China	22.82 (3.23) 22.00		41.96 (19.90) 35.00		19.14 (20.08) 12.00	
Having a first child (male)	The United States	24.19 (3.90) 25.00	Wald χ² = 32.12, *B* = −2.11***	44.41 (9.94) 44.00	Wald χ² = 36.74, *B* = 6.99***	20.23 (11.06) 19.00	Wald χ² = 50.75, *B* = 9.11***
	China	26.13 (3.52) 26.00		39.94 (10.33) 40.00		13.80 (10.80) 11.00	
Having a first child (female)	The United States	23.01 (3.54) 23.00	Wald χ² = 27.84, *B* = −1.88***	40.48 (8.98) 40.00	Wald χ² = 33.67, *B* = 6.39***	17.47 (9.19) 17.00	Wald χ² = 55.00, *B* = 8.27***
	China	24.55 (3.23) 25.00		35.45 (7.57) 35.00		10.90 (7.42) 10.00	

*Note.* **p < .01. ***p < .001.

#### The Role of Filial Piety

Then, as part of our exploratory analyses, we examined whether cultural differences in the widths of the time windows could be explained by beliefs in filial piety . To do so, we conducted a series of regression analyses, controlling for the same covariates.

When the outcome variable was the time window for career-related life events, culture predicted the time window (β = −.59, *b* = −24.78 [−28.43, −21.13], *p* < .001) and filial piety beliefs (β = .45, *b* = 0.62 [0.50, 0.74], *p* < .001). When culture and filial piety beliefs were entered simultaneously as predictors, beliefs in filial piety predicted a narrower time window for career-related life events (β = −.26, *b* = −7.83 [−10.10, −5.56], *p* < .001).

When the outcome variable was the time window for family-related life events, culture predicted the time window (β = −.51, *b* = −15.40 [−18.12, −12.68], *p* < .001) and filial piety beliefs (*β* = .45, *b* = 0.62 [0.50, 0.74], *p* < .001). When culture and filial piety beliefs were entered simultaneously as predictors, beliefs in filial piety predicted a narrower time window for family-related life events (β = −.30, *b* = −6.60 [−8.27, −4.92], *p* < .001). Taken together, these analyses show that filial piety is positively associated with the social clock’s rigidity, and support the idea that cultural differences in the social clock’s rigidity are due to cultural differences in the endorsement of filial piety beliefs.

#### Anticipated Negative Emotional Outcomes of Deviation From the Social Clock (Upper Bound)

**Figure 2.**
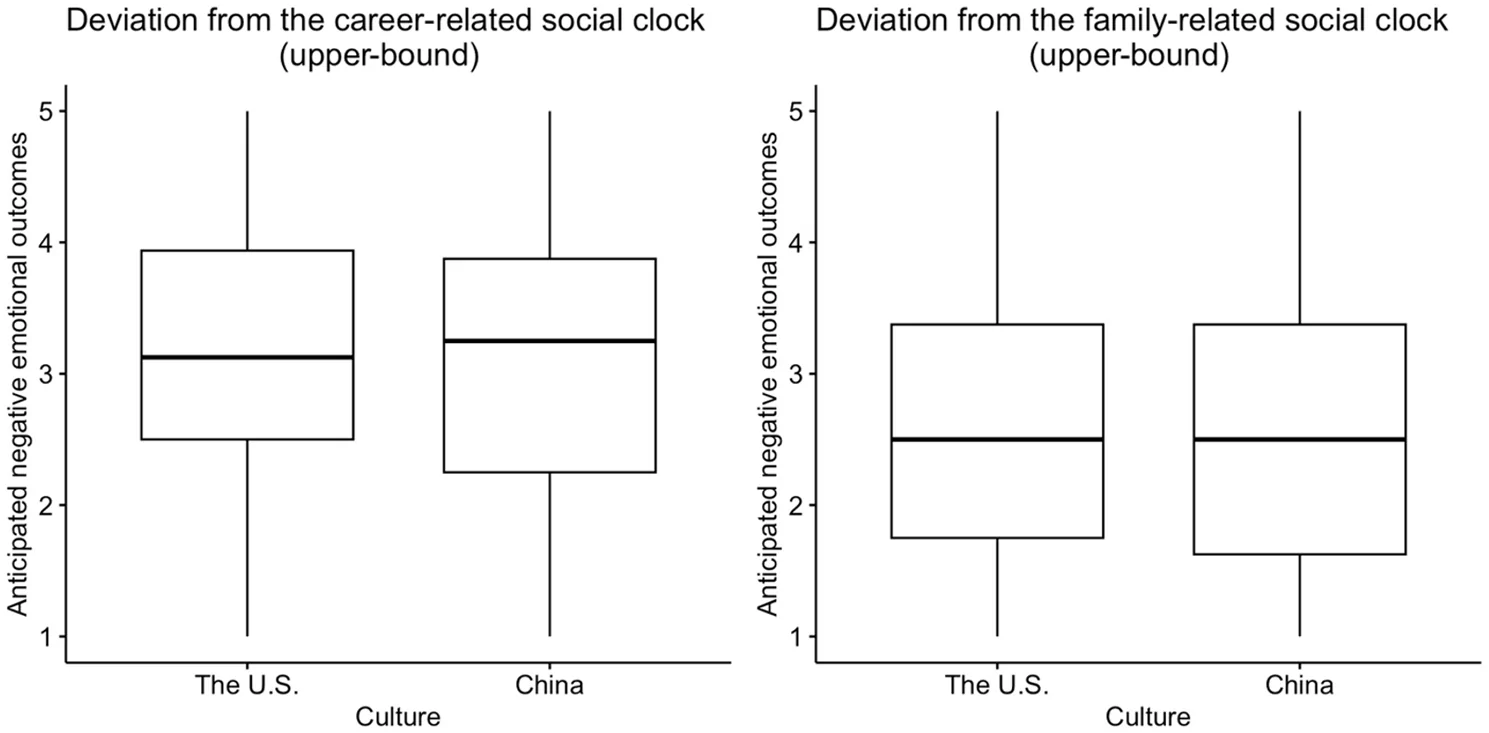
Anticipated negative emotional outcomes of deviation from the social clock (upper bound) in the United States versus China (Study 1a).

In addition, we examined whether there are cultural differences in anticipated emotional outcomes resulting from deviation from the upper bound of the social clock (Figure 2). The analyses showed no cultural differences. See full results in the supplementary materials (p. 6).

### Discussion

Study 1a provided the initial evidence about how the social clock’s rigidity differs between the two national cultures. Consistent with our hypothesis, the social clock was more flexible in the United States than in China, as the Chinese social clock has significantly higher lower bound and lower upper bound ages compared to the American social clock. Not surprisingly, the cultural differences in the social clock were primarily driven by the differences in the upper bound age, perhaps reflecting the fact that lower bound ages are more narrowly restricted by biological and structural (e.g., length of typical formal schooling) factors. We also found initial support that these differences were explained by cultural differences in the endorsement of filial piety beliefs. However, contrary to our hypothesis, deviation from the upper bound of the social clock resulted in comparable anticipated negative emotional outcomes in both cultures. We reason that this may be because the participants were mostly young adults (*M*_age_ = 22.88, *SD*_age_ = 4.91) who may not yet have real-life experiences with failing to achieve specific life milestones within the appropriate time windows. In addition, the American participants were more ethnically diverse than the national demographics, which may dilute the cultural main effect. Therefore, in Study 1b, we recruited participants with a wider age range who also more accurately reflect the national ethnic breakdown.

## Study 1b

Study 1b (preregistered at https://aspredicted.org/7R8_GXN) aimed to replicate the findings of Study 1a and to increase the external validity with participants from a wider age range.

### Participants

Based on power analysis,^5^ we set our sample size at *N* = 100 per culture. We recruited 107 participants in the United States via the Prolific platform and 102 participants in China via the Credamo platform (comparable to the Prolific platform). The data exclusion rules were the same as in Study 1a. Our final sample (*N* = 198) includes 98 American participants and 100 Chinese participants (Table 5).

**Table 5.** Breakdown of Demographics in the United States and China in Study 1b.

Culture	Age *M* (*SD*)	Gender (%)	Ethnicity (%)	College degree (%)
The United States (*N* = 98)	34.80 (11.10)	Male: 50 Female: 44.9 Nonbinary/Other: 5.1	European American: 72.4 Asian American/Pacific Islander: 10.2 Hispanic/Latino: 8.2 Black/African American: 7.1 Multiracial/Biracial: 2.0	63.3
China (*N* = 100)	34.59 (12.73)	Male: 52.0 Female: 48.0	Han Chinese: 98.0 Ethnic Minorities: 2.0	96.0

### Measures

The procedure and measures were the same as in Study 1a, except that we removed all additional measures that were not central to the hypothesis. We averaged the ratings of the four anticipated negative emotions into a single composite score in each time window block (*α*s > .88). Filial piety beliefs were measured using the same scale (Fu et al., 2020; α_US_ = .89; α_CH_ = .77).

We deviated from the preregistration in that the intersubjective perceptions of the social clock and anticipated negative emotional outcomes of deviation from the intersubjective social clock (upper bound) were not included in this paper. These measures are identical to those in Study 1a. We did not report the preregistered analyses related to the intersubjective perceptions of the social clock, as those will be presented in a separate paper (Zang & Kim, in preparation). In addition, we dropped the event of beginning graduate school when finalizing study materials, as it is not a common life milestone.

### Results

#### Correlations Between Variables

We calculated the descriptive statistics of the variables and bivariate correlations between them in the United States and China (Table 6).

**Table 6.** Descriptive Statistics of the Variables and Bivariate Correlations Between Them in the United States and China in Study 1b.

	The United States (*N* = 98)								China (*N* = 100)							
Variable	*M* (*SD*)	1	2	3	4	5	6	7	*M* (*SD*)	1	2	3	4	5	6	7
1. Time window for career-related life events	43.00 (18.69)	—							9.75 (10.09)	—						
2. Time window for family-related life events	34.73 (14.22)	.62***	—						10.58 (6.52)	.51***	—					
3. Filial piety beliefs	3.12 (.67)	−.28**	−.34***	—					3.94 (.44)	−.23*	−.19	—				
4. Anticipated negative emotional outcomes resulting from deviation from the career-related social clock (upper bound deviation)	2.60 (1.09)	−.33***	−.23*	.11	—				3.59 (.90)	−.17	−.09	.34***	—			
5. Anticipated negative emotional outcomes resulting from deviation from the family-related social clock (upper bound deviation)	2.14 (.99)	−.20*	−.16	.36***	.58***	—			3.25 (.97)	−.08	−.11	.36***	.61***	—		
6. Age	34.80 (11.10)	.10	−.03	.37***	−.32**	−.03	—		34.59 (12.73)	−.14	−.01	.04	−.19	−.01	—	
7. SES	4.85 (1.66)	.08	.13	−.01	−.05	.01	.07	—	5.33 (1.16)	−.03	.21*	.17	.10	.08	.18	—

*Note.* SES = socioeconomic status.

* *p* < .05. **p < .01. ***p < .001.

#### Cultural Differences in the Time Windows

**Figure 3.**
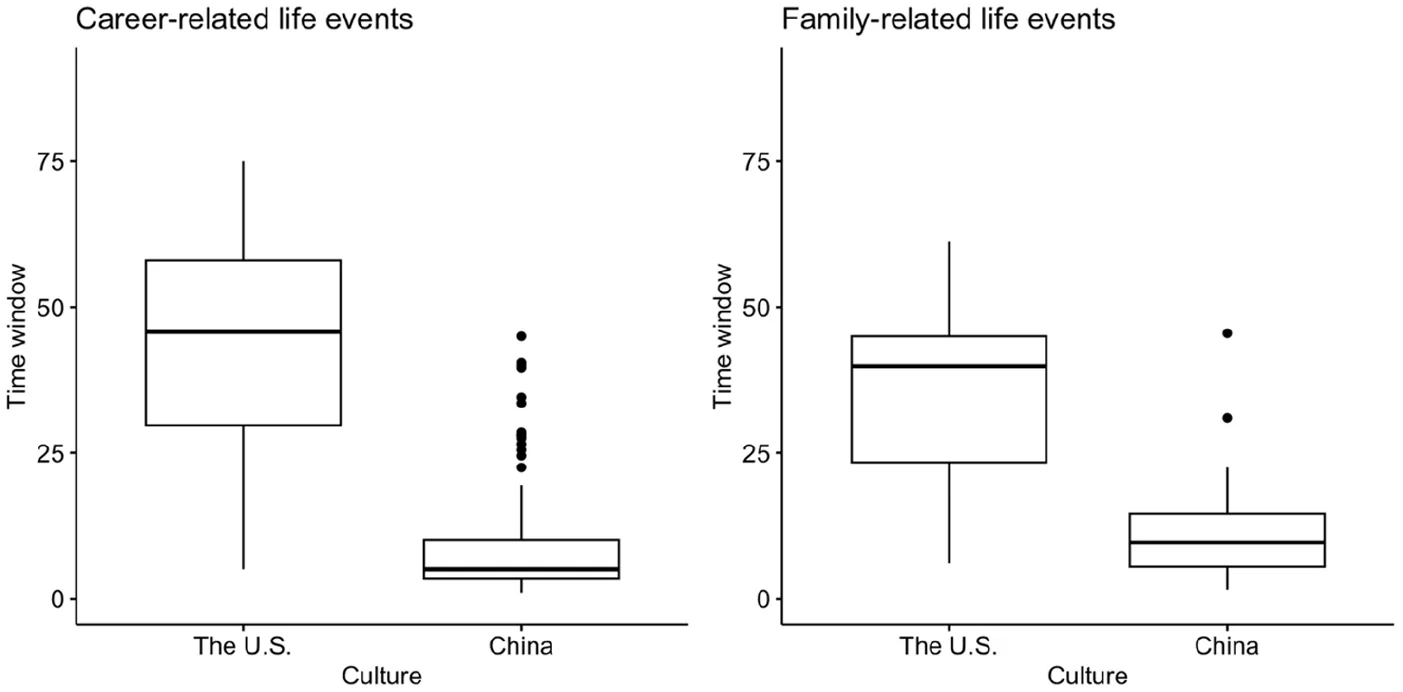
The widths of the time windows for career-related and family-related life events in the United States and China in Study 1b.

Likewise, we examined whether culture affects the social clock’s rigidity (Figure 3). In all analyses, we included the same demographic covariates as in Study 1a.

We first examined the cultural differences in the time window for career-related life events. A GEE with robust SEs was conducted on the time window for career-related life events with culture (the United States vs. China) as a predictor with the covariates.^6^ We found a significant main effect of culture,^7^ Wald χ²(1, *N* = 198) = 226.92, *p* < .001. The time window for career-related life events was narrower in China (*M* = 9.69, robust *SE* = 1.12, 95% CI [7.49, 11.89]) than in the United States (*M* = 43.06, robust *SE* = 1.81, [39.51, 46.61]), *B* = 33.37, robust *SE* = 2.22, [29.03, 37.71].

We ran the same analysis for the cultural differences in the time window for family-related life events. Similarly, we found a significant main effect of culture, Wald χ²(1, *N* = 198) = 260.13, *p* < .001. The time window for family-related life events was narrower in China (*M* = 10.37, robust *SE* = 0.68, 95% CI [9.03, 11.71]) than in the United States (*M* = 34.95, robust *SE* = 1.34, [32.32, 37.58]), *B* = 24.58, robust *SE* = 1.52, [21.59, 27.56]. As in Study 1a, we found significant cultural differences in most of the lower and upper bound ages when examined separately, although the magnitude of the differences was greater for the upper bound ages (Table 7).^8^

**Table 7.** Descriptive Statistics and Cultural Differences in the Time Window for Each Life Event, as well as in the Lower and Upper Bound Age in Study 1b (Controlled for Demographic Covariates).

Specific life event	Culture	Lower bound age Mean (*SD*) Median	Wald χ² (1, *N* = 198) and B for the effect of culture on the lower bound age	Upper bound age Mean (*SD*) Median	Wald χ²(1, *N* = 198) and B for the effect of culture on the upper bound age	Time window Mean (*SD*) Median	Wald χ²(1, *N* = 198) and B for the effect of culture on the time window
Starting college	The United States	17.19 (1.44) 17.00	Wald χ² = 3.35, *B* = −.48	64.70 (21.15) 61.00	Wald χ² = 176.71, *B* = 36.77***	47.51 (21.55) 45.00	Wald χ² = 175.04, *B* = 37.24***
	China	17.70 (1.91) 18.00		28.51 (14.28) 22.00		10.81 (14.66) 4.00	
Getting a full-time job	The United States	18.08 (2.34) 18.00	Wald χ² = 76.59, *B* = −3.34***	56.57 (20.85) 61.00	Wald χ² = 136.51, *B* = 26.16***	38.49 (21.48) 44.00	Wald χ² = 166.04, *B* = 29.50***
	China	21.59 (2.68) 22.00		30.28 (7.73) 28.00		8.69 (7.99) 6.00	
Getting married (male)	The United States	22.46 (4.06) 22.00	Wald χ² = 11.04, *B* = −1.76***	73.41 (19.72) 80.00	Wald χ² = 343.79, *B* = 38.70 ***	50.95 (21.28) 60.50	Wald χ² = 337.52, *B* = 40.46***
	China	24.24 (2.72) 24.50		35.74 (8.57) 35.00		11.50 (8.65) 9.00	
Getting married (female)	The United States	21.36 (3.54) 21.00	Wald χ² = 10.01, *B* = −1.58**	69.26 (23.49) 80.00	Wald χ² = 203.66, *B* = 36.40***	47.90 (23.82) 59.00	Wald χ² = 217.16, *B* = 37.98***
	China	22.94 (2.87) 22.00		33.75 (9.84) 31.00		10.81 (9.96) 9.00	
Having a first child (male)	The United States	24.29 (4.26) 25.00	Wald χ² = 5.48, *B* = −1.28*	46.11 (11.25) 45.00	Wald χ² = 46.22, *B* = 9.71***	21.83 (12.58) 20.00	Wald χ² = 50.88, *B* = 10.98***
	China	25.80 (2.89) 26.00		36.52 (6.36) 35.00		10.72 (6.37) 10.00	
Having a first child (female)	The United States	22.71 (3.94) 22.50	Wald χ² = 9.11, *B* = −1.47**	40.98 (7.90) 40.00	Wald χ² = 65.21, *B* = 7.44***	18.27 (8.56) 17.50	Wald χ² = 76.93, *B* = 8.91***
	China	24.09 (2.75) 24.00		33.38 (5.03) 32.00		9.29 (5.38) 8.50	

*Note.* *p < .05. **p < .01. ***p < .001.

#### The Role of Filial Piety

Then, as exploratory analyses, we examined whether the cultural differences in the widths of the time windows could be explained by beliefs in filial piety. We conducted a series of regression analyses, controlling for the covariates.

When the outcome variable was the time window for career-related life events, culture predicted the time window (β = −.75, *b* = −33.37 [−37.71, −29.03], *p* < .001) and filial piety beliefs (β = .59, *b* = 0.82 [0.66, 0.99], *p* < .001). When culture and filial piety beliefs were entered simultaneously as predictors, beliefs in filial piety predicted a narrower time window for career-related life events (β = −.21, *b* = −6.67 [−10.32, −3.03], *p* < .001).

When the outcome variable was the time window for family-related life events, culture predicted the time window (β = −.75, *b* = −24.58 [−27.73, −21.44], *p* < .001) and filial piety beliefs (β = .59, *b* = 0.82 [0.66, 0.99], *p* < .001). When culture and filial piety beliefs were entered simultaneously as predictors, beliefs in filial piety predicted a narrower time window for family-related life events (β = −.23, *b* = −5.44 [−8.06, −2.83], *p* < .001). Taken together, these analyses suggest that filial piety is positively associated with the social clock’s rigidity, and present further support for the notion that cultural differences in the social clock’s rigidity are due to cultural differences in the endorsement of filial piety beliefs.

#### Anticipated Negative Emotional Outcomes of Deviation From the Social Clock (Upper Bound)

**Figure 4.**
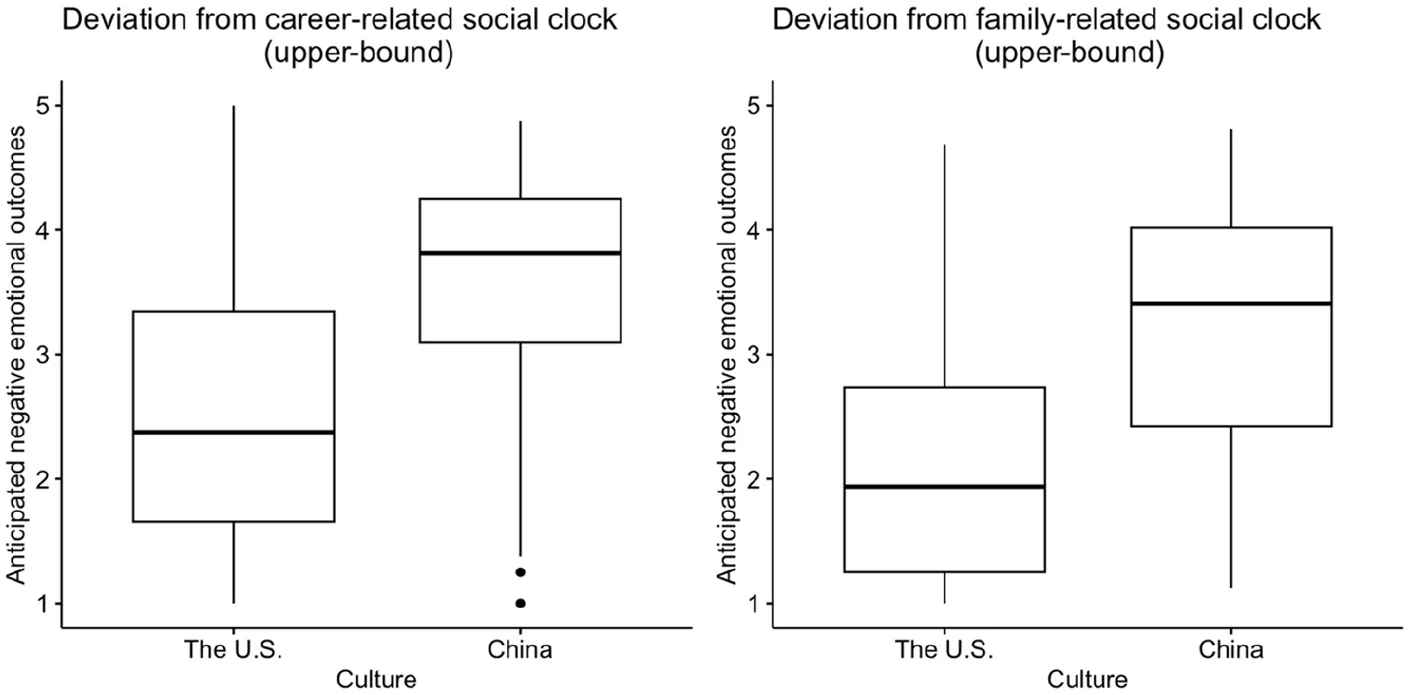
Anticipated negative emotional outcomes of deviation from the social clock (upper bound) in the United States versus China (Study 1b).

As an exploratory analysis, we further examined whether anticipated negative emotional outcomes resulting from deviation from the upper bound of the social clock differ across cultures (Figure 4).

We first examined the anticipated negative emotional outcomes resulting from deviation from the career-related social clock. A GEE with robust SEs was conducted on this variable with culture (the United States vs. China) as a predictor with the covariates. We found a significant main effect of culture, Wald χ²(1, *N* = 198) = 43.62, *p* < .001. Deviation from the career-related social clock (upper bound) was anticipated to be more distressing in China (*M* = 3.57, robust *SE* = 0.09, 95% CI [3.39, 3.75]) than in the United States (*M* = 2.62, robust *SE* = 0.10, [2.41, 2.82]), *B* = −0.95, robust *SE* = 0.14, [−1.23, −0.67]).

In addition, we tested anticipated negative emotional outcomes resulting from deviation from the family-related social clock. Similarly, we found a significant main effect of culture, Wald χ²(1, *N* = 198) = 50.76, *p* < .001. Deviation from the family-related social clock was also anticipated to be more distressing in China (*M* = 3.23, robust *SE* = 0.10, 95% CI [3.03, 3.42]) than in the United States (*M* = 2.16, robust *SE* = 0.10, [1.96, 2.36]), *B* = −1.07, robust *SE* = 0.15, [−1.36, −0.77]. See the Supplemental Material (p. 10) for descriptive statistics and the cultural differences in anticipated negative emotional outcomes resulting from deviation from the social clock (upper bound) for each life event.

#### The Role of Filial Piety

Then, we examined whether the cultural differences in anticipated negative emotional outcomes resulting from deviation from the upper bound of the social clock could be explained by beliefs in filial piety as exploratory analyses. To do so, we conducted a series of regression analyses, controlling for the same covariates.

With deviation from the career-related social clock, culture predicted anticipated negative emotional outcomes (β = .43, *b* = 0.95 [0.66, 1.24], *p* < .001) and filial piety beliefs (β = .59, *b* = 0.82 [0.66, 0.99], *p* < .001). When culture and filial piety were entered simultaneously as predictors, beliefs in filial piety predicted more anticipated negative emotional outcomes (β = .30, *b* = 0.47 [0.23, 0.71], *p* < .001).

With deviation from the family-related social clock, culture predicted anticipated negative emotional outcomes (β = .48, *b* = 1.07 [0.77, 1.36], *p* < .001) and filial piety beliefs (β = .59, *b* = 0.82 [0.66, 0.99], *p* < .001). When culture and filial piety were entered simultaneously as predictors, beliefs in filial piety predicted more anticipated negative emotional outcomes (β = .40, *b* = 0.65 [0.41, 0.88], *p* < .001). These analyses show that filial piety is positively associated with anticipated negative emotions, and that cultural differences in the endorsement of filial piety beliefs are likely to explain the cultural differences in anticipated negative emotional outcomes.

### Discussion

Study 1b replicated Study 1a findings that the social clock tends to be more rigid in China than in the United States and that cultural differences in endorsement of filial piety beliefs seem to play an important role. In addition, unlike Study 1a, we found that deviation from the upper bound of the social clock was anticipated to be more distressing for Chinese than Americans, despite that Americans’ average upper age bound is higher than that of Chinese and that such cultural differences were also meaningfully related to cultural differences in the endorsement of filial piety beliefs. We reason that this is because we recruited participants with an older average age who may have more real-life experience (either direct or vicarious) regarding failure to achieve specific life milestones within the appropriate time windows. In addition, we recruited a sample in the United States that better represents the national demographics. As such, we believe that Study 1b is more likely to represent the actual cultural differences in how deviation from the upper bound of the social clock affects emotional well-being.

## Study 2

The present research examines the cultural differences in the rigidity of the social clock and anticipated emotional well-being associated with achieving important life milestones beyond age-appropriate expectations. Yet, Studies 1a and 1b only examined the psychological consequences of deviation from the upper bound of the social clock. Given that achieving life milestones too early can also cause distress, as individuals may feel unprepared or socially out of sync, Study 2 aimed to provide a more thorough understanding of culture and the social clock by further examining how deviation from the lower bound of the social clock would affect emotional well-being. We hypothesized that, in addition to replicating the results regarding the time window differences, deviation from the social clock (lower bound) would be anticipated to be more distressing for the Chinese than for American participants, and that this cultural difference would be explained by cultural differences in the endorsement of filial piety beliefs. The study was preregistered (https://doi.org/10.17605/OSF.IO/45BUW).^9^

### Participants

Based on power analysis,^10^ we set our sample size at *N* = 100 per culture. We recruited 100 participants in the United States via the Prolific platform and 111 participants in China via the Naodao platform (comparable to the Prolific platform). The data exclusion rules were the same as in Studies 1a and 1b. Our final sample (*N* = 201) includes 92 American participants and 109 Chinese participants (Table 8).

**Table 8.** Breakdown of Demographics in the United States and China in Study 2.

Culture	Age *M* (*SD*)	Gender (%)	Ethnicity (%)	College degree (%)
The United States (*N* = 92)	35.17 (9.36)	Male: 44.6 Female: 54.3 Nonbinary/Other: 1.1	European American: 51.1 Black/African American: 27.2 Asian American/Pacific Islander: 7.6 Hispanic/Latino: 5.4 Multiracial/Biracial: 4.3 Native American/Alaskan: 3.3 Other: 1.1	58.7
China (*N* = 109)	25.07 (4.89)	Male: 62.4 Female: 37.6	Han Chinese: 99.1 Ethnic Minorities: 0.9	95.4

### Measures

The procedures and measures were the same as in Studies 1a and 1b, except that we measured anticipated negative emotional outcomes of deviation from the lower bound of the social clock. We averaged the ratings of the four anticipated negative emotions into a single composite score in each time window block (αs > .80). Filial piety beliefs were measured using the same scale (Fu et al., 2020; α_US_ = .92; α_CH_ = 0.79). The study also included additional measures examining the subjective (i.e., how participants would judge lower bound social clock deviation) and intersubjective negative social judgments of deviation from the social clock (i.e., how participants think a typical person in their country would judge lower bound social clock deviation). In addition, the study included the measures of the intersubjective perceptions of the social clock and anticipated negative emotional outcomes of deviation from the lower bound of the intersubjective social clock. Finally, we included the tightness/looseness scale (Gelfand et al., 2011) for an exploratory purpose. See the supplementary materials for a full description of these measures (pp. 11–13).

### Results

#### Correlations Between Variables

Likewise, we calculated the descriptive statistics of the variables and bivariate correlations between them in the United States and China (Table 9).

**Table 9.** Descriptive Statistics of the Variables and Bivariate Correlations Between Them in the United States and China in Study 2.

	The United States (*N* = 92)									China (*N* = 109)								
Variable	*M* (*SD*)	1	2	3	4	5	6	7	8	*M* (*SD*)	1	2	3	4	5	6	7	8
1. Time window for career-related life events	38.97 (18.66)	—								9.26 (8.50)	—							
2. Time window for family-related life events	31.81 (14.17)	.64***	—							9.04 (6.75)	.55***	—						
3. Filial piety beliefs	3.29 (.75)	−.53***	−.32**	—						4.11 (.45)	−.25**	−.27**	—					
4. Tightness/Looseness	3.32 (.50)	−.03	−.11	.16	—					4.01 (.34)	−.23*	−.16	.30**	—				
5. Anticipated negative emotional outcomes of deviation from the career-related social clock (lower bound)	1.95 (.72)	−.01	.02	−.09	.14	—				1.94 (.84)	−.08	−.07	.23*	.05	—			
6. Anticipated negative emotional outcomes of deviation from the family-related social clock (lower bound)	2.09 (.84)	.05	.05	−.004	.001	.34***	—			2.36 (.93)	−.07	.14	.12	.31**	.55***	—		
7. Age	35.17 (9.36)	.02	−.01	.04	−.16	.03	−.23 *	—		25.07 (4.89)	−.05	−.10	.01	−.15	.03	−.15	—	
8. SES	5.03 (1.65)	−.06	−.10	.10	.01	−.06	.01	.21*	—	5.19 (1.24)	−.08	−.14	.16	.06	.26**	.10	.24*	—

*Note.* SES = socioeconomic status.

* *p* < .05. **p < .01. ***p < .001.

Given that the primary purpose of Study 2 was to examine how deviation from the lower bound of the social clock would affect emotional well-being, we included the replication results for cultural differences in the time windows and the role of filial piety in explaining the cultural differences in the supplementary materials (pp. 13–19).^11^

#### Anticipated Negative Emotional Outcomes of Deviation From the Social Clock (Lower Bound)

**Figure 5.**
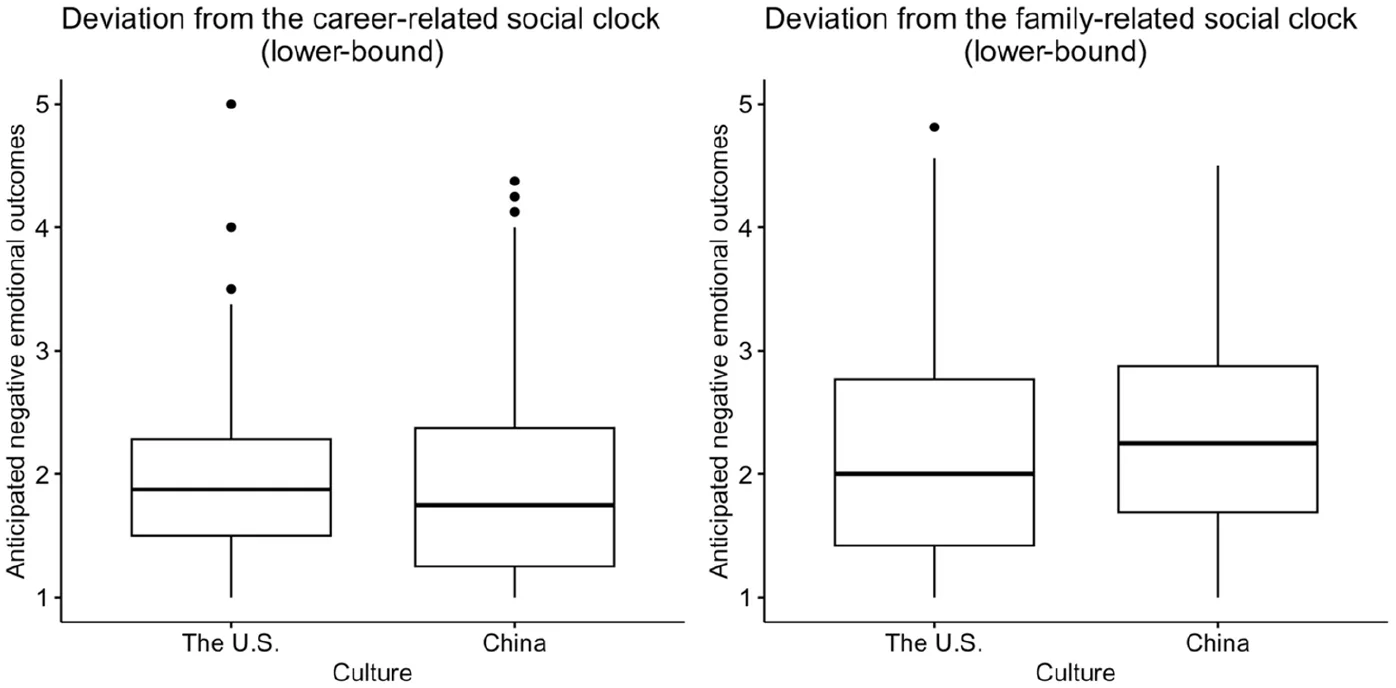
Anticipated negative emotional outcomes of deviation from the social clock (lower bound) in the United States versus China (Study 2).

We examined whether anticipated emotional outcomes resulting from deviation from the social clock differ across cultures (Figure 5). In all analyses, we included age, gender, SES, and education as covariates.

We first looked examined the anticipated negative emotional outcomes resulting from deviation from the career-related social clock. A GEE with robust SEs was conducted on the anticipated negative emotional outcomes with culture (the United States vs. China) as a predictor with the covariates did not reveal a significant main effect of culture, Wald χ²(1, *N* = 201) = .10, *p* = .76. Anticipated negative emotional outcomes of deviation from the career-related social clock did not significantly differ between the United States (*M* = 1.97, robust *SE* = 0.08, 95% CI [1.80, 2.13]) and China (*M* = 1.93, robust *SE* = 0.08, [1.77, 2.09]), *B* = 0.04, robust *SE* = 0.12, [−0.20, 0.28].

Then, we repeated the analysis with anticipated negative emotional outcomes resulting from deviation from the family-related social clock. Similarly, we did not find a significant main effect of culture, Wald χ²(1, *N* = 201) = .002, *p* = .96. Anticipated negative emotional outcomes of deviation from the family-related social clock did not significantly differ between the United States (*M* = 2.24, robust *SE* = 0.09, 95% CI [2.05, 2.42]) and China (*M* = 2.24, robust *SE* = 0.09, [2.06, 2.42]), *B* = −-0.007, robust *SE* = 0.14, [−0.28, 0.27].

### Exploratory Analysis With Tightness/Looseness

**Figure 6.**
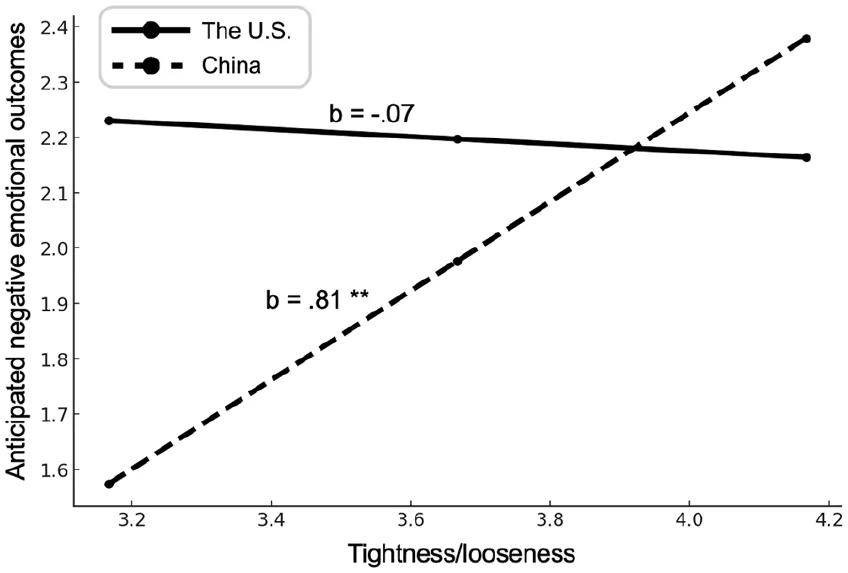
Association between tightness/looseness and anticipated negative emotional outcomes of deviation from the family-related social clock in the United States and China. *Note.* Simple slopes are unstandardized. **p < .01.

Unlike the first two studies, we did not find strong correlations between filial piety and emotional outcomes. Notably, we found a positive correlation between tightness/looseness and anticipated negative emotions of deviation from the family-related social clock among the Chinese participants (*r*(107) = .310, *p* = .001) but not among the American participants (*r*(90) = .001, *p* = .991). This suggests that culture might moderate the relationship between tightness/looseness and anticipated negative emotions. Thus, as outlined in our preregistration, we conducted exploratory analyses to examine the role of tightness/looseness (Gelfand et al., 2011) in anticipated negative emotions. A moderation analysis was conducted using the PROCESS macro in SPSS (Hayes, 2018) to examine whether the relationship between tightness/looseness and anticipated negative emotions of deviation from the family-related social clock was moderated by culture with the covariates,^12^ *b* = 0.87 [0.27, 1.47], *SE* = 0.31, *t*(193) = 2.85, *p* = .005. The overall model indicates that the predictors collectively accounted for 11.4% of the variance in anticipated negative emotions, *R*^2^ = .114, *F* (7, 193) = 3.55, *p* = .001. Simple slopes analysis suggests that there was a positive association between tightness/looseness and anticipated negative emotions in China [simple *b* = 0.81 [0.32, 1.29], *SE* = 0.25, *t*(193) = 3.25, *p* = .001], but no significant association in the United States [simple *b* = −0.07 [−0.43, 0.29], *SE* = 0.18, *t*(193) = −0.36, *p* = .72] (Figure 6). That is, in China, those with low tolerance for deviant behaviors anticipated feeling worse than those with greater tolerance, whereas in the United States, they did not differ from their counterparts in anticipated emotions.

### Discussion

Study 2 replicated the findings that the social clock tends to be more rigid in China than in the United States and that filial piety beliefs appear to play an important role. However, deviation from the lower bound of the social clock did not differ in emotional outcomes between the two national cultures, and filial piety was not or only weakly correlated with the outcomes. It may be the case that such deviation, unlike deviation from the upper bound, still fulfills the duties to the family by achieving milestones, and thus, the Chinese do not consider it particularly problematic either.

Interestingly, exploratory analysis suggested that tightness/looseness was positively associated with anticipated negative emotions resulting from deviation from the family-related social clock in China, but not in the United States. We should be cautious about drawing any conclusion, but it raises the possibility that the social clock is so flexible in the United States that deviation from it (at least from the lower bound) is not considered a violation of a social norm, unlike in China. We did not find such interaction for negative emotional outcomes resulting from deviation from the career-related social clock. This may be attributed to the perception that early attainment of career-related life milestones signals competence, and is therefore less likely to elicit negative emotions. Taken together, this study advances our understanding of why and how the two cultures differ in their responses to the social clock violation.

## General Discussion

### Summary

Across three studies, we found that the social clock was more rigid in China than in the United States, and the results support the idea that these differences are explained by cultural differences in filial piety. These findings show that China, which is more collectivistic than the United States, tends to have tighter social clocks due to differences in the value placed on interpersonal duties and responsibilities, specifically, responsibilities toward one’s parents and elders (i.e., filial piety; Schwartz et al., 2010).

We also examined how deviation from the social clock is associated with anticipated emotional well-being. Study 1b, with a broader age range, supported our hypothesis that upper bound deviation from the social clock would be anticipated to be more distressing for the Chinese than American participants, with these differences explained by cultural differences in filial piety. Despite the mean upper bound age being higher in the United States, the Chinese reported more anticipated negative emotional outcomes. It is also worth noting that quite a few American participants gave the highest number permitted (i.e., 90) when asked to provide an upper bound age, suggesting that they may not believe such an age limit exists. Thus, the upper bound ages may be less meaningful for Americans than for the Chinese, which further supports our finding that the social clock tends to be more flexible in the United States. This pattern differed in anticipated distress resulting from being too early on the social clock in Study 2 both in terms of the lack of cultural difference in emotions and the potential underlying reasons as discussed above. It is notable that different cultural beliefs may underlie cultural differences in anticipated emotional responses to deviation from the upper and lower bounds of the social clock.

### Theoretical Contribution and Societal Implication

Our findings contribute to the literature on societal expectations about ages for specific life milestones (e.g., Helson & McCabe, 1994; Lee & Payne, 2010; Peterson, 1996). These previous studies primarily focused on the average age at which people engage in life events and provided demographic and economic explanations. The current research, which examines societal expectations about age and life events, suggests a novel social psychological explanation—filial piety, a specific form of social duty. Leveraging cultural differences in the importance of fulfilling social duties, we showed that the value of social duties underlies individuals’ endorsement of the social clock. Our studies also show the specificity of such cultural differences. The results from Study 2 show that social clock deviation that does not compromise fulfilling social duties did not lead to cultural differences in anticipated negative emotions and was not strongly correlated with filial piety. This implies that cultural differences in the social clock’s rigidity are distinct from those in general permissibility of participating in non-age-sensitive events (e.g., learning to ride a bicycle), which are less likely tied to social obligations.

Methodologically, the present research is the first to extend the point estimates (i.e., mean ages, or mean upper bound and lower bound ages; e.g., Peterson, 1996) to the interval estimates (i.e., free-response time windows beyond fixed intervals) of the age expectations for achieving specific life milestones across cultures. This methodology allows us to examine both when social clocks are set and how rigid social clocks are in different societies.

The current research also advances the understanding of timings in the human life cycle, which is governed by both the biological clock (e.g., Friese et al., 2006; Leader, 2006) and the social clock. One of the biological clock examples is the pressure to have a child during one’s most fertile years (Leader, 2006). Unlike biological clocks, which are roughly universal across cultures due to neural-physical development, the current study suggests that the social clock tends to vary across cultures. Therefore, the present research advances our understanding of timings in the human life cycle by identifying a culturally dependent element (i.e., the social clock) in addition to the culturally constant element (i.e., the biological clock). Furthermore, the current study contributes to research on cultural life scripts, which primarily examine culturally unique and shared life events as well as their typical timing and order of occurrence (e.g., Hatiboğlu & Habermas, 2016; Ottsen & Berntsen, 2013) by focusing not only on the list of events and their sequence in life memory stories but also on the intensity of age-related pressure people experience throughout their lives across cultures.

The findings of the current research could have significant societal implications. On one hand, a more rigid social clock may lead to undesirable social consequences. For instance, a more rigid social clock in China echoes pervasive age discrimination in the Chinese job market, where companies sometimes limit the age of applicants to younger than 35 years. This discrimination may lead to the loss of experienced employees and a lack of innovation due to limited career mobility. Moreover, an extremely rigid social clock may backfire, causing generational rifts and societal problems when the younger generation “rebels” against strong social norms, either due to pragmatic or ideological reasons, such as the cases in Korea and Japan with the low birth rates among younger generations (e.g., Yun et al., 2022). On the other hand, a more rigid social clock may be collectively adaptive in certain cultures as it may help stabilize employment and birth rates by motivating people to remain employed and have children within a specific time window. The current study may prompt policymakers in societies with rigid age expectations to examine whether, behind the back of a tighter social clock, there lie more intensive social issues.

### Limitations and Future Directions

The current study focused only on two cultures, which limits the generalizability of its findings to more cultures. Thus, future researchers could consider implementing the study across more cultures (using demographically comparable and larger samples) to examine whether the social clock’s rigidity systematically varies along the individualism–collectivism dimension, along with pertinent explanatory psychological constructs. We reason that other specific types of social duty (e.g., familismo, a central Latinx cultural value, see Ayón et al., 2010) or religious duty (e.g., Judaism religious duties, see Cohen et al., 2013, for the religion and spirituality of Jews) may be relevant among non-East Asian collectivistic cultures.

Moreover, we focused on a small subset of frequently mentioned life events based on prior research on cultural life scripts (e.g., Hatiboğlu & Habermas, 2016; Ottsen & Berntsen, 2013). Although we believe that our findings would apply to other unmeasured important life events, further research on additional life events is needed to confirm the generalizability of the observed patterns.

In addition, future research could investigate the interpersonal consequences of deviating from the social clock. Compared to people from more individualistic cultures (e.g., the United States), people from more collectivistic cultures (e.g., China) may judge targets whose personal life timing deviates from the social clock more negatively, and these more negative judgments may, in turn, influence social interactions. We thus encourage future research to investigate interpersonal or social consequences of deviation from the social clock.

Another noticeable limitation that arises in cross-cultural studies is that measures may not be precisely equivalent due to linguistic and cultural differences and, consequently, a possible breach of measurement invariance. Future studies could implement a priori Exploratory Factor Analysis to ensure measurement equivalence. Also, future research could employ experimental approaches of cultural priming with bicultural participants, manipulating cultural contexts or key psychological constructs, such as filial piety, to further support causal interpretations of cultural differences in the rigidity of the social clock.

Finally, the current study measured anticipated negative emotional outcomes, instructing participants to assume that they wanted to achieve certain life milestones yet failed to; however, it is possible that people may not experience comparable negative self-conscious emotions if they have no desire to attain specific life milestones. Future research could investigate whether people who fail to abide by the social clock due to more uncontrollable reasons (e.g., financial restraints) versus more controllable reasons (e.g., personal choice) differ in their emotional distress resulting from deviation from the social clock. Moreover, actual negative emotional outcomes of deviation from the social clock may differ from those that were anticipated, and those of deviation from the social clock (completing life milestones too early or too late) may differ from those associated with life events never achieved (e.g., Luhmann et al., 2021). Future research could use longitudinal or cross-sectional analyses with larger samples to examine whether anticipated well-being outcomes align with actual emotional responses, as well as how missed life events affect emotional well-being.

## Conclusion

The present study suggests that people’s perceptions of age-related social expectations, and in particular, the rigidity (or flexibility) of the social clock, could be shaped by the culture in which we reside. The current study aims to offer a clue to the puzzle of where the urge to achieve important life milestones by a particular age originates. We believe a better understanding of how and why culture shapes the social clock would be beneficial in the pursuit of “following what one’s heart desired, without transgressing what was right” (adapted from Confucius, 1999).

## Supplemental Material

sj-docx-1-psp-10.1177_01461672251362514 – Supplemental material for Culture and the Social Clock: Cultural Differences in the Optimal Timing of Life Supplemental material, sj-docx-1-psp-10.1177_01461672251362514 for Culture and the Social Clock: Cultural Differences in the Optimal Timing of Life by Lu Zang and Heejung Kim in Personality and Social Psychology Bulletin

sj-docx-2-psp-10.1177_01461672251362514 – Supplemental material for Culture and the Social Clock: Cultural Differences in the Optimal Timing of Life Supplemental material, sj-docx-2-psp-10.1177_01461672251362514 for Culture and the Social Clock: Cultural Differences in the Optimal Timing of Life by Lu Zang and Heejung Kim in Personality and Social Psychology Bulletin

## Notes

1. These previous studies had a sample size of 130 to 141 per study. We oversampled in this exploratory study to enhance reliability by doubling the sample size per culture.

2. See the supplemental materials (p. 6) for the post hoc power analyses using the G*Power software (Erdfelder et al., 1996).

3. We ran GEEs with robust SEs for all analyses (except for regression analyses) in Study 1a to maintain consistency and account for violations of key statistical assumptions (e.g., homogeneity of variance) in the outcome variables. The significance patterns of all reported results in Study 1a remain consistent with or without demographic covariates and will not be discussed further.

4. We controlled for the lower bound ages in examining the cultural differences in the time windows, and the main effect of culture remained significant. Specifically, we first examined the cultural differences in the time window for career-related life events. A GEE with robust SEs on the time window for career-related life events with culture (the United States vs. China) as a predictor, controlling for demographic covariates and the lower bound age for career-related life events revealed a significant main effect of culture, Wald χ²(1, *N* = 640) = 118.20, *p* < .001. We then ran the same analysis for the cultural differences in the time window for family-related life events, controlling for demographic covariates and the lower bound age for family-related life events. Similarly, we found a significant main effect of culture, Wald χ²(1, *N* = 640) = 59.79, *p* < .001. We are unable to control for the upper bound ages due to high correlations between time windows and upper bound ages, which would result in problematic multicollinearity (with *r*s > .98) if both were included in the same GEE model.

5. Based on the results of Study 1a (cultural differences on time windows), the medium *R* = 0.52, we calculated the effect size *f*^2^ = 0.38. We ran the power analysis using the G*Power software (Erdfelder et al., 1996) using an *F* test, A Priori, where *f*^2^ = 0.38, α = .05, Power (1 − β) = .95, number of predictors = 3. It yields a total sample size of 50. We oversampled to increase statistical power and double the minimum sample size needed per culture. See the Supplemental Material (p. 7) for the corrected details and post hoc power analyses.

6. We had preregistered to conduct univariate ANOVAs to examine the cultural differences, but we decided to instead run GEEs with robust SEs for all analyses (except for regression analyses) in Study 1b to account for violations of key statistical assumptions in the outcome variables. See the Supplemental Material (pp. 7–9) for the ANOVA results as preregistered.

7. The significance patterns of all reported results in Studies 1b remain consistent with or without demographic covariates and will not be discussed further.

8. We controlled for the lower bound ages in examining the cultural differences in the time windows, and the main effect of culture remained significant. Specifically, we first examined the cultural differences in the time window for career-related life events. A GEE with robust SEs on the time window for career-related life events with culture (the United States vs. China) as a predictor, controlling for demographic covariates and the lower bound age for career-related life events revealed a significant main effect of culture, Wald χ²(1, *N* = 198) = 146.21, *p* < .001. We then ran the same analysis for the cultural differences in the time window for family-related life events, controlling for demographic covariates and the lower bound age for family-related life events. Similarly, we found a significant main effect of culture, Wald χ²(1, *N* = 198) = 163.85, *p* < .001. We are unable to control for the upper bound ages due to high correlations between time windows and upper bound ages, which would result in problematic multicollinearity (with *r*s > .98) if both were included in the same GEE model.

9. We deviated from the preregistration such that other measures, the intersubjective perceptions of the social clock and judgment of deviation from the social clock, were not included as a part of this paper.

10. Based on the results of Study 1b (culture and filial piety simultaneously predicting anticipated negative emotional outcomes), we ran the power analysis using the G*Power software (Erdfelder et al., 1996) using an *F* test, a priori, where the smallest *R*^2^ = 0.308, *f*^2^ = 0.445, α = .05, Power (1 − β) = .80, number of predictors = 6. It yields a total sample size of 38. We opted for a fivefold oversampling to increase statistical power. See the supplemental materials (pp. 10–11) for the post hoc power analyses.

11. Since we preregistered mediation analyses with filial piety, we also include mediation analysis results using the PROCESS macro (Hayes, 2018).

12. We conducted a post hoc power analysis using the G*Power software (Erdfelder et al., 1996) to compute the achieved power of the moderation analysis, given α (.05), sample size (*N* = 201), number of tested predictors (1, i.e., the interaction term), total number of predictors (7), and effect size (*f*^2^ = 0.04). The achieved power is 0.82, indicating that the study had sufficient power to detect the observed moderation effect.
