# Supplementary material for: Culture and the Social Clock: Cultural Differences in the Optimal Timing of Life
Source: Pers Soc Psychol Bull. 2025 Sep 1;52(10):3144–63. doi: 10.1177/01461672251362514 (PMC13507491; doi:10.1177/01461672251362514)
Supplement: Supplementary file 1 [file sj-docx-1-psp-10.1177_01461672251362514.docx]

**Supplementary Materials for Culture and the Social Clock:**

**Cultural Differences in the Optimal Timing of Life**

In the Supplementary Materials, we report additional measures and results for Studies 1a, 1b, and 2.

**Supplementary Materials for Study 1a**

**Study 1a Additional Measures and Results**

***Intersubjective Perceptions of the Social Clock***

We also measured participants’ perceptions of the intersubjective social clock by asking them to provide the lower- and upper-bound ages they thought *a typical person* in their country would consider appropriate for engaging in each life event. For instance, participants read: “Assuming a person wants to attend a college. What would *a typical American person* think about the appropriate age range for the person to start attending college?” Then, participants were instructed to scroll two sliders (ranging from 10 to 90) to indicate the lower- and upper-bound ages they thought a typical American person would think appropriate for the person to start attending college, respectively.

***Anticipated Negative Emotional Outcomes of Deviation from the Intersubjective Social Clock (Upper-bound)***

We measured the anticipated negative self-conscious emotions resulting from deviation from the upper-bound of the intersubjective social clock. For example, participants read: “Assuming you want to attend a college—Imagine you didn’t start attending college by the time you are reaching *this upper-bound* of the appropriate age for attending college (i.e., by age [quoting the numeric value of the upper-bound age of the intersubjective social clock they entered previously]). Please indicate how much you would feel the following emotions if you are not fulfilling the age-appropriate expectation.” Similarly, participants were presented with four emotional states, i.e., “regretful,” “ashamed,” “anxious,” and “guilty,” and reported how much they would feel each emotion on a 5-point Likert-type scale (1 = *not at all* to 5 = *a great deal*). The structure and phrasing of questions were comparable in all blocks.

These two measures were presented immediately following each “perceptions of the social clock” question within the six time window blocks.

At the end of each time window block, we asked participants to indicate how much they agreed or disagreed with the following statements on a 5-point Likert-type scale (1 = *strongly disagree*, 5 = *strongly agree*). The questions in the first time window block were “It would be easy for me to start attending college even if I am older than the typical college students (*M* = 2.72, *SD* = 1.03)” and “I will not be judged based on my age if I start attending college later than the typical college students (*M* = 2.60, *SD* = 1.07).” The questions in the second time window block were “It would be easy for me to get a full-time job even if I am older than the typical full-time job seekers (*M* = 2.86, *SD* = 1.06)” and “I will not be judged based on my age if I start applying for full-time jobs later than the typical full-time job seekers (*M* = 2.68, *SD* = 1.12).” The questions in the third and fourth time window block were “Suppose I were a male (or a female), it would be easy for me to get married at an age later than the typical age by which males (or females) generally get married (*M* supposing one is a male = 3.24, *SD* supposing one is a male = 1.09; *M* supposing one is a female = 2.89, *SD* supposing one is a female = 1.17)” and “Suppose I were a male (or a female), I will not be judged based on my age if I get married later than the typical age by which males (or females) generally get married (*M* supposing one is a male = 3.03, *SD* supposing one is a male = 1.13; *M* supposing one is a female = 2.40, *SD* supposing one is a female = 1.19).” The questions in the fifth and sixth time window block were “Suppose I were a male (or a female), it would be easy for me to have a child at an age later than the typical age by which males (or females) generally had their first child (*M* supposing one is a male = 3.23, *SD* supposing one is a male = 1.11; *M* supposing one is a female = 2.61, *SD* supposing one is a female = 1.17)” and “Suppose I were a male (or a female), I will not be judged based on my age if I decide to have a child later than the typical age by which males (or females) generally had their first child (*M* supposing one is a male = 2.92, *SD* supposing one is a male = 1.11; *M* supposing one is a female = 2.31, *SD* supposing one is a female = 1.13).”

Besides, after demographic questions, we asked participants to answer “yes” or “no” on whether they had a college degree (*N* yes = 283, 44.8%; *N* no = 350, 54.7%; *N* system-missing = 3, 0.5%), had a full-time job (*N* yes = 215, 33.6%; *N* no = 425, 66.4%), were married (*N* yes = 66, 10.3%; *N* no = 573, 89.5%; *N* system-missing = 1, 0.2%), and had one or more kids (*N* yes = 55, 8.6%; *N* no = 585, 91.4%). We also asked participants to indicate how much they agreed or disagreed that they were “on-the-clock”^[1]^ in terms of attending college (*M* = 4.77, *SD* = 1.41), getting a full-time job (*M* = 4.11, *SD* = 1.52), getting married (*M* = 3.31, *SD* = 1.67), and having their first child (*M* = 3.01, *SD* = 1.67) on a 5-point Likert-type scale (1 = *strongly disagree*, 5 = *strongly agree*).

In addition, we measured participants’ motivational systems using the BIS/BAS scale (Carver & White, 1994) and their inclinations to use cooperative or competitive strategies using the cooperative/competitive strategy scale (Simmons et al., 1988) as alternate explanatory psychological mechanisms. These measures occurred after the filial piety scale (Fu et al., 2020) and before demographic information collection.

As exploratory analyses, we examined if the cultural differences in the widths of the time windows could be explained by the Behavioral Inhibition System (BIS) and Behavioral Activation System (BAS) motivational systems (Carver & White, 1994) and inclinations to use cooperative or competitive strategies (Simmons et al., 1988). See Table 1 for the simultaneous regression analysis predicting the career-related and family-related time windows.

We first examined the time window for career-related life events. A simultaneous multiple regression analysis was conducted to identify the predictors of the time window for career-related life events. We examined seven predictors: culture, filial piety, BIS, BAS, competitive strategy use, cooperative strategy use, and competitive strategy avoidance, along with demographic covariates. Together, these variables explained 36.2% of the variance in the time window for career-related life events, *R*^2^ = .36; *F* (11, 628) = 32.44, *p* < .001. Among the predictors, culture (*β* = -.47, *p* < .001) and filial piety (*β* = -.19, *p* < .001) emerged as the strongest predictors, while competitive strategy avoidance was also significant (*β* = -.10, *p* = .013). The remaining predictors were not associated with the time window for career-related life events (*p*s > .05).

We then examined the time window for family-related life events. Similarly, we conducted a simultaneous multiple regression analysis with the seven predictors to examine their association with the time window for family-related life events, along with demographic covariates. Together, these variables explained 33.4% of the variance in the time window for career-related life events, *R*^2^ = .33; *F* (11, 628) = 28.61, *p* < .001. Similarly, among the predictors, culture (*β* = -.37, *p* < .001) and filial piety (*β* = -.22, *p* < .001) emerged as the strongest predictors, while competitive strategy avoidance was also significant (*β* = -.12, *p* = .003). The remaining predictors were not associated with the time window for family-related life events (*p*s > .05). In sum, even with the inclusion of these additional factors, filial piety remained to be a significant predictor for the time windows. In addition, competitive strategy avoidance independently predicted time windows for career-related and family-related life events, suggesting that a stronger motivation to avoid competitive strategy and their successful outcomes is associated with narrower time windows. Future research could further explore the role of competitive strategy avoidance.

**Table 1**

*Simultaneous regression analysis predicting the time windows for* *career-related and family-related life events.*

| Predictor variable/Outcome variable | Time window for career-related life events | | | | |
| --- | --- | --- | --- | --- | --- |
|  | *b* | 95 % CI for *b* | *β* | *t*(628*)* | *p* |
| Culture | -19.91 | [-23.82, -15.99] | -.47 | -9.99 | < .001 |
| Filial piety | -5.68 | [-8.33, -3.04] | -.19 | -4.23 | < .001 |
| BIS | -.75 | [-3.57, 2.07] | -.02 | -.53 | .600 |
| BAS | .56 | [-3.29, 4.42] | .01 | .29 | .775 |
| Competitive strategy use | -2.33 | [-5.10, .43] | -.06 | -1.66 | .098 |
| Cooperative strategy use | -.71 | [-3.52, 2.10] | -.02 | -.50 | .619 |
| Competitive strategy avoidance | -3.08 | [-5.49, -.66] | -.10 | -2.50 | .013 |
|  | Time window for family-related life events | | | | |
|  | *b* | 95 % CI for *b* | *β* | *t*(628*)* | *p* |
| Culture | -11.29 | [-14.16, -8.42] | -.37 | -7.72 | < .001 |
| Filial piety | -4.80 | [-6.74, -2.86] | -.22 | -4.87 | < .001 |
| BIS | -1.62 | [-3.68, .45] | -.06 | -1.53 | .125 |
| BAS | .20 | [-2.62, 3.03] | .01 | .14 | .888 |
| Competitive strategy use | -1.91 | [-3.94, .12] | -.07 | -1.85 | .066 |
| Cooperative strategy use | .69 | [-1.38, 2.75] | .02 | .65 | .515 |
| Competitive strategy avoidance | -2.70 | [-4.47, -.93] | -.12 | -3.00 | .003 |

**Anticipated Negative Emotional Outcomes of Deviation from the Social Clock (Upper-bound)**

We first looked at anticipated negative emotional outcomes resulting from deviation from the career-related social clock. A GEE with robust SEs was conducted^[2]^ on the anticipated negative emotional outcomes with culture (the U.S. vs. China) as a predictor, and age, gender, SES, and education as covariates did not reveal a significant main effect of culture, Wald χ²(1, *N* = 640) = .35, *p* = .55 (*M_US_* = 3.07, robust SE*_US_* = .06, 95% Wald CI*_US_* [2.95, 3.20]; *M_CH_* = 3.13, robust SE*_CH_* = .07, 95% Wald CI*_CH_* [3.00, 3.27]), B = -.06, robust SE = .10, 95% Wald CI [-.26, .14].

Then, we repeated the same analysis with anticipated negative emotional outcomes resulting from deviation from the family-related social clock. Similarly, we did not find a significant main effect of culture, Wald χ²(1, *N* = 640) = 2.70, *p* = .10 (*M_US_* = 2.49, robust SE*_US_* = .06, 95% Wald CI*_US_* [2.37, 2.62]; *M_CH_* = 2.66, robust SE*_CH_* = .07, 95% Wald CI*_CH_* [2.53, 2.80]), B = -.17, robust SE = .11, 95% Wald CI [-.38, .03].

**Post Hoc Power Analysis**

We ran a post hoc power analysis using the G*Power software (Erdfelder et al., 1996) based on Study 1a results (culture and filial piety simultaneously predicting the time windows), given *α* (.05), sample size (*N* = 640), number of predictors (6), and effect size (smallest *f*^2^ = .458). The achieved power is approximately 1.00, indicating a very high probability of detecting a significant effect if it exists.

**Supplementary Materials for Study 1b**

**Power Analysis for Study 1b**

Our original way of conducting the power analysis did not take into account filial piety when predicting the time windows. Based on the Study 1a results (culture and filial piety simultaneously predicting the time windows), we ran another corrected power analysis after collecting data using the G*Power software (Erdfelder et al., 1996) using an *F* test, A Priori, where the smallest *R*^2^ = .314, *f*^2^ = .458, *α* = .05, Power (1- *β*) = .80, number of predictors = 6. It yields a total sample size of 37. It suggests that we had a fivefold oversampling and obtained adequate statistical power.

In addition, we ran a post hoc power analysis to compute achieved power (based on Study 1b results regarding culture and filial piety simultaneously predicting anticipated negative emotional outcomes), given α (.05), sample size (*N* = 198), number of predictors (6), and effect size (smallest *f*^2^ = .445). The achieved power is approximately 1.00, indicating a very high probability of detecting a significant effect if it exists.

**Study 1b Additional Results**

***Cultural Differences in the*** ***Time Windows***

**ANOVA Results.** We first examined the cultural differences in the time window for career-related life events. A univariate ANOVA on the time window for career-related life events with culture (the U.S. vs. China) as a fixed factor and age, gender, SES, and education as covariates revealed a significant main effect of culture, *F* (1, 192) = 230.35, *p* < .001, *η*^2^_p_ = .55 [.45, .62] (*M*_American_ = 43.00, *SD*_American_ = 18.69; *M*_Chinese_ = 9.75, *SD*_Chinese_ = 10.09). We then examined the cultural differences in the time window for family-related life events. We ran a univariate ANOVA on the time window for family-related life events with culture (the U.S. vs. China) as a fixed factor and age, gender, SES, and education as covariates. Similarly, we found a significant main effect of culture, *F* (1, 192) = 237.63, *p* < .001, *η*^2^_p_ = .55 [.46, .62] (*M*_American_ = 34.73, *SD*_American_ = 14.22; *M*_Chinese_ = 10.58, *SD*_Chinese_ = 6.52) (see Table 2).

**Table 2**

*Descriptive statistics and* *pairwise comparisons for each life event in Study 1b.*

| Specific life event | Culture | Lower bound age  Mean (SD) | Upper bound age  Mean (SD) | Time window Mean (SD) | Pairwise comparison |
| --- | --- | --- | --- | --- | --- |
| Starting college | The U.S. | 17.19 (1.44) | 64.70 (21.15) | 47.51 (21.55) | *t* = 13.69, *p* < .001 |
|  | China | 17.70 (1.91) | 28.51 (14.28) | 10.81 (14.66) |  |
| Getting a full-time job | The U.S. | 18.08 (2.34) | 56.57 (20.85) | 38.49 (21.48) | *t* = 12.42, *p* < .001 |
|  | China | 21.59 (2.68) | 30.28 (7.73) | 8.69  (7.99) |  |
| Getting married (male) | The U.S. | 22.46 (4.06) | 73.41 (19.72) | 50.95 (21.28) | *t* = 17.38, *p* < .001 |
|  | China | 24.24 (2.72) | 35.74 (8.57) | 11.50 (8.65) |  |
| Getting married (female) | The U.S. | 21.36 (3.54) | 69.26 (23.49) | 47.90 (23.82) | *t* = 14.48, *p* < .001 |
|  | China | 22.94 (2.87) | 33.75 (9.84) | 10.81 (9.96) |  |
| Having a first child (male) | The U.S. | 24.29 (4.26) | 46.11 (11.25) | 21.83 (12.58) | *t* = 7.31, *p* < .001 |
|  | China | 25.80 (2.89) | 36.52 (6.36) | 10.72 (6.37) |  |
| Having a first child (female) | The U.S. | 22.71 (3.94) | 40.98 (7.90) | 18.27 (8.56) | *t* = 8.41, *p* < .001 |
|  | China | 24.09 (2.75) | 33.38 (5.03) | 9.29  (5.38) |  |

***Anticipated Negative Emotional Outcomes of Deviation from the Social Clock***

**ANOVA Results.** As an exploratory analysis, we examined whether there are cultural differences in anticipated emotional outcomes resulting from deviation from the social clock. We first looked at negative emotional outcomes resulting from deviation from the career-related social clock. A univariate ANOVA on negative emotions resulting from deviation from the career-related social clock with culture (the U.S. vs. China) as a fixed factor and age, gender, SES, and education as covariates revealed a significant main effect of culture, *F* (1, 192) = 42.00, *p* < .001, *η*^2^_p_ = .18 [.09, .27] (*M*_American_ = 2.60, *SD*_American_ = 1.09; *M*_Chinese_ = 3.59, *SD*_Chinese_ = .90). In addition, we tested negative emotional outcomes resulting from deviation from the family-related social clock using a univariate ANOVA on negative emotional outcomes resulting from deviation from the family-related social clock with culture (the U.S. vs. China) as a fixed factor and age, gender, SES, and education as covariates. Similarly, we found a significant main effect of culture, *F* (1, 192) = 51.36, *p* < .001, *η*^2^_p_ = .21 [.12, .31] (*M*_American_ = 2.14, *SD*_American_ = .99; *M*_Chinese_ = 3.25, *SD*_Chinese_ = .97).

**Table 3**

*Descriptive statistics and pairwise comparisons between anticipated emotional outcomes of deviation from the social clock (upper bound) in the U.S. and China in Study 1b.*

| Specific life event | The U.S. | China | Pairwise comparison |
| --- | --- | --- | --- |
| Starting college | 2.40 (1.16) | 3.39 (1.00) | *t* = -5.84, *p* < .001 |
| Getting a full-time job | 2.79 (1.38) | 3.79 (.96) | *t* = -5.51, *p* < .001 |
| Getting married (male) | 2.07 (1.11) | 3.28 (1.01) | *t* = -7.27, *p* < .001 |
| Getting married (female) | 2.24 (1.18) | 3.17 (1.16) | *t* = -4.79, *p* < .001 |
| Having a first child (male) | 2.04 (1.08) | 3.33 (1.03) | *t* = -7.96, *p* < .001 |
| Having a first child (female) | 2.22 (1.20) | 3.22 (1.15) | *t* = -5.42, *p* < .001 |

***Psychological Outcomes (GEE)***

**Table 4**

*Descriptive statistics and the* *cultural differences in* *anticipated negative emotional outcomes* *resulting from* *deviation from the* *social clock (upper bound) for each life event (controlling for demographic covariates).*

| Specific life event | The U.S.  Mean (SD) | China  Mean (SD) | Wald χ²(1, *N* = 198) and B for the effect of culture on anticipated negative emotional outcomes resulting from deviation from the social clock (upper bound) |
| --- | --- | --- | --- |
| Starting college | 2.40 (1.16) | 3.39 (1.00) | Wald χ² = 32.57, B = -.93 *** |
| Getting a full-time job | 2.79 (1.38) | 3.79 (.96) | Wald χ² = 31.00, B = -.97 *** |
| Getting married (male) | 2.07 (1.11) | 3.28 (1.01) | Wald χ² = 52.09, B = -1.17 *** |
| Getting married (female) | 2.24 (1.18) | 3.17 (1.16) | Wald χ² = 21.95, B = -.84 *** |
| Having a first child (male) | 2.04 (1.08) | 3.33 (1.03) | Wald χ² = 62.22, B = -1.28 *** |
| Having a first child (female) | 2.22 (1.20) | 3.22 (1.15) | Wald χ² = 30.67, B = -.97 *** |

*** *p* < .001.

**Supplementary Materials for Study 2**

**Power Analysis for Study 2**

To maintain transparency, we retained our previous power analyses using the G*Power software (Erdfelder et al., 1996) in the main manuscript, as they reflect the original methods. The statistics in these analyses were identical to the correct power analysis based on the Study 1b results (culture and filial piety simultaneously predicting anticipated negative emotional outcomes).

In addition, we ran a post hoc power analysis to compute achieved power (based on Study 2 results regarding culture and filial piety simultaneously predicting time windows), given α (.05), sample size (*N* = 201), number of predictors (6), and effect size (smallest *f*^2^ = 1.37). The achieved power is approximately 1.00, indicating a very high probability of detecting a significant effect if it exists.

**Study 2 Additional Measures and Results**

***Subjective Negative Social Judgments of Deviation from the Social Clock***

We measured subjective negative social judgments of deviation from the social clock. For instance, participants read: “Assuming there is a person who wants to attend a college—Imagine that person started attending college before the time they reach this lower bound of the appropriate age for attending college (i.e., before age [quoting the numeric value of the lower-bound age of the subjective social clock they entered previously]). Please indicate how much you think that person SHOULD feel the following emotions if they are not fulfilling this age-appropriate expectation.” Participants were presented with four emotional states, i.e., “regretful,” “ashamed,” “anxious,” and “guilty,” and reported how much they thought that person *should* feel each emotion on a 5-point Likert-type scale (1 = *not at all* to 5 = *a great deal*) (Zang & Kim, 2024).

***Intersubjective Negative Social Judgments of Deviation from the Social Clock***

After that, we measured intersubjective negative social judgments of deviation from the social clock. For example, participants read: “Recap: there is a person who wants to attend a college but did so before the time they reach this lower bound of the appropriate age for attending college (i.e., before age [quoting the same numeric value of the lower-bound age of the subjective social clock they entered previously]). To your best guess, please indicate how much *a typical American person* would think that person SHOULD feel the following emotions if they are not fulfilling this age-appropriate expectation.” Participants were presented with the same four emotional states and reported how much they thought a typical American person would think that person *should* feel each emotion on a 5-point Likert-type scale (1 = *not at all* to 5 = *a great deal*). The structure and phrasing of questions were comparable in all blocks.

The two negative social judgment measures were presented before each “intersubjective perceptions of the social clock” question within the six time window blocks.

***Intersubjective Perceptions of the Social Clock and Anticipated Negative Emotional Outcomes of Deviation from the Intersubjective Social Clock (Lower-bound)***

Similar to Studies 1a and 1b, we measured participants’ perceptions of the intersubjective social clock and anticipated negative emotions of deviations from the lower-bound of the intersubjective social clock. The procedures and measures were identical to the additional measures in Studies 1a and 1b, except that we focused on anticipated negative emotions of deviation from the lower-bound of the intersubjective social clock.

These procedures and measures were preregistered for a separate research project (<https://osf.io/r36ky/?view_only=6e277d48d4844f1fa3cbbafd870d5de3>). Likewise, we did not report the preregistered analyses related to the intersubjective perceptions of the social clock, given that these measures and their associated results, along with those from Studies 1a and 1b, will be reported in a separate research paper (Zang & Kim, in preparation).

***Tightness/Looseness***

The strength of perceived social norms and tolerance of deviation was measured by the tightness-looseness scale (Gelfand et al., 2011) with the sample items: “There are many social norms that people are supposed to abide by in this country,” “People in this country have a great deal of freedom in how they want to behave in most situations (reverse),” and “In this country, if someone acts in an inappropriate way, others will strongly disapprove (Gelfand et al., 2011)”. Participants indicated how much they agreed or disagreed with each statement on a 5-point Likert-type scale (1 = *strongly disagree*, 5 = *strongly agree*). The ratings of the six items were averaged into a composite score (*M*_American_ = 3.32, *S.D.*_American_ = .50, *α* = 0.51; *M*_Chinese_ = 4.01, *SD*_Chinese_ = .34, *α* = 0.36); higher numbers indicated stronger perceptions that social norms are “pervasive, clearly defined, and reliably imposed within nations” (Gelfand et al., 2011).

***Cultural Differences in the Time Windows***

We had preregistered to conduct univariate ANOVAs to examine whether culture affects the social clock’s rigidity (Figure 1), but ultimately, we decided to instead run GEEs with robust SEs for all analyses to account for violations of key statistical assumptions (e.g., homogeneity of variance) in the outcome variables. (except for mediation analyses) in Study 2 to account for violations of key statistical assumptions (e.g., homogeneity of variance) in the outcome variables. Below, we report both ANOVA and GEE results.

**Figure 1**

*The widths of the time windows for career-related and family-related life events in the U.S. and China in Study 2.*

**ANOVA Results.** We first examined the cultural differences in the time window for career-related life events. A univariate ANOVA on the time window for career-related life events with culture (the U.S. vs. China) as a fixed factor and age, gender, SES, and education as covariates revealed a significant main effect of culture, *F* (1, 195) = 120.56, *p* < .001, *η*^2^_p_ = .38 [.28, .47] (*M*_American_ = 38.97, *SD*_American_ = 18.66; *M*_Chinese_ = 9.26, *SD*_Chinese_ = 8.50). We then examined the cultural differences in the time window for family-related life events. We ran a univariate ANOVA on the time window for family-related life events with culture (the U.S. vs. China) as a fixed factor and age, gender, SES, and education as covariates. Similarly, we found a significant main effect of culture, *F* (1, 195) = 129.12, *p* < .001, *η*^2^_p_ = .40 [.30, .48] (*M*_American_ = 31.81, *SD*_American_ = 14.17; *M*_Chinese_ = 9.04, *SD*_Chinese_ = 6.75) (see Table 5).

**Table 5**

*Descriptive statistics and pairwise comparisons for each life event in Study 2.*

| Specific life event | Culture | Lower bound age  Mean (SD)  Median | Upper bound age  Mean (SD)  Median | Time window Mean (SD)  Median | Pairwise comparison |
| --- | --- | --- | --- | --- | --- |
| Starting college | The U.S. | 17.39 (1.49)  17.00 | 58.34 (23.77)  57.00 | 40.95 (24.06)  40.50 | *t* = 9.34, *p* < .001 |
|  | China | 18.02 (1.45)  18.00 | 27.43 (10.06)  23.00 | 9.41 (10.01)  6.00 |  |
| Getting a full-time job | The U.S. | 18.95 (2.87)  18.00 | 55.93 (18.94)  60.00 | 36.99 (19.98)  42.00 | *t* = 9.12, *p* < .001 |
|  | China | 22.57 (2.64)  23.00 | 31.67 (9.91)  29.00 | 9.10 (10.57)  6.00 |  |
| Getting married (male) | The U.S. | 23.71 (4.24)  24.00 | 67.43 (21.85)  70.00 | 43.73 (23.04)  47.50 | *t* = 10.07, *p* < .001 |
|  | China | 24.15 (2.47)  25.00 | 34.65 (9.84)  32.00 | 10.50 (10.24)  8.00 |  |
| Getting married (female) | The U.S. | 22.82 (3.81)  23.00 | 66.92 (22.96)  70.00 | 44.11 (23.50)  49.50 | *t* = 10.56, *p* < .001 |
|  | China | 23.03 (2.37)  23.00 | 32.50 (9.31)  30.00 | 9.48 (9.32)  7.00 |  |
| Having a first child (male) | The U.S. | 25.09 (4.18)  25.00 | 46.29 (10.57)  45.00 | 21.21 (12.13)  20.00 | *t* = 7.98, *p* < .001 |
|  | China | 26.03 (2.38)  26.00 | 34.20 (5.65)  34.00 | 8.17 (5.99)  6.00 |  |
| Having a first child (female) | The U.S. | 23.52 (3.68)  23.50 | 41.73 (8.95)  40.00 | 18.21 (9.47)  16.50 | *t* = 7.71, *p* < .001 |
|  | China | 24.34 (2.70)  25.00 | 32.35 (4.71)  32.00 | 8.01 (5.16)  6.00 |  |

**GEE Results.** We first examined the cultural differences in the time window for career-related life events. A GEE with robust standard errors was conducted on the time window for career-related life events with culture (the U.S. vs. China) as a predictor and age, gender, SES, and education as covariates. We found a significant main effect of culture, Wald χ²(1, *N* = 201) = 97.41, *p* < .001. The time window for career-related life events was narrower in China (*M* = 10.06, robust SE = 1.14, 95% Wald CI [7.83, 12.30]) than in the U.S. (*M* = 38.01, robust SE = 2.21, 95% Wald CI [33.68, 42.34]), B = 29.95, robust SE = 2.83, 95% Wald CI [22.40, 33.50].

We then examined the cultural differences in the time window for family-related life events. We ran a GEE with robust standard errors on the time window for family-related life events with culture (the U.S. vs. China) as a predictor and age, gender, SES, and education as covariates. Similarly, we found a significant main effect of culture, Wald χ²(1, *N* = 201) = 101.57, *p* < .001. The time window for family-related life events was narrower in China (*M* = 9.36, robust SE = .86, 95% Wald CI [7.67, 11.05]) than in the U.S. (*M* = 31.44, robust SE = 1.71, 95% Wald CI [28.08, 34.79]), B = 22.08, robust SE = 2.19, 95% Wald CI [17.78, 26.37]. See Table 6 for descriptive statistics and cultural differences in the time window for each life event, as well as in the lower and upper bound age. As in Studies 1a and 1b, we found significant cultural differences in most of the lower- and upper-bound ages when examined separately, although the magnitude of the differences was greater with the upper-bound ages^[3]^.

**Table 6**

*Descriptive statistics and cultural differences in the time window for each life event, as well as in the lower and upper bound age in Study 2 (controlled for demographic covariates).*

***The Role of Filial Piety***

Although we report analyses using multiple regressions in the main text, we report the results for the role of filial piety in Study 2 using a mediational analysis approach as pre-registered. We conducted a mediation analysis using the ordinal least squares regression (path analysis) via the PROCESS macro in SPSS (Hayes, 2018), in which filial piety beliefs mediate the effect of culture on the time windows, controlling for the covariates (Figure 2).

**Figure 2**

*The mediation results in Study 2 (N = 201). Total effects are shown in parentheses. Path coefficients are standardized regression coefficients (βs). Since X (Culture) is categorical (0 = U.S., 1 = China), the indirect effect is partially standardized, with M (Filial Piety Belief) and Y (Career- or Family-related Time Window) standardized. *** p < .001.*

When the outcome variable was the time window for career-related life events, culture predicted the time window (*β* = -.68, *b* = -27.95 [-32.97, -22.93], *p* < .001) and filial piety beliefs (*β* = .52, *b* = .77 [.56, .98], *p* < .001). Chinese participants were more likely to view the time window for career-related life events as narrower and to endorse beliefs in filial piety. Beliefs in filial piety, in turn, predicted a narrower time window for career-related life events (*β* = -.37, *b* = -10.38 [-13.41, -7.35], *p* < .001). A bootstrap confidence interval (based on 5,000 samples) for the partially standardized indirect effect did not include zero, 95% CI [-.54, -.25], although after controlling for filial piety beliefs, there was still a significant direct association between culture and the time window for career-related life events (*β* = -.49, *b* = -19.99 [-25.08, -14.91], *p* < .001). Thus, filial piety beliefs partially mediated the cultural difference in the time window for career-related life events, as predicted.

When the outcome variable was the time window for family-related life events, culture predicted the time window (*β* = -.70, *b* = -22.08 [-25.91, -18.25], *p* < .001) and filial piety beliefs (*β* = .52, *b* = .77 [.56, .98], *p* < .001). Chinese participants were more likely to view the time window for family-related life events as narrower and to endorse beliefs in filial piety. Beliefs in filial piety, in turn, predicted a narrower time window for family-related life events (*β* = -.23, *b* = -4.93 [-7.40, -2.45], *p* < .001). A bootstrap confidence interval (based on 5,000 samples) for the partially standardized indirect effects did not include zero, 95% CI [-.39, -.10], although after controlling for filial piety beliefs, there was still a significant direct association between culture and the time window for family-related life events (*β* = -.58, *b* = -18.30 [-22.46, -14.15], *p* < .001). Thus, filial piety beliefs partially mediated the cultural difference in the time window for family-related life events, as predicted.

***Anticipated Negative Emotional Outcomes of Deviation from the Social Clock (Lower Bound)***

**ANOVA Results.** We examined whether there are cultural differences in anticipated emotional outcomes resulting from deviation from the social clock. We first looked at negative emotional outcomes resulting from deviation from the career-related social clock. A univariate ANOVA on negative emotions resulting from deviation from the career-related social clock with culture (the U.S. vs. China) as a fixed factor and age, gender, SES, and education as covariates did not reveal a significant main effect of culture, *F* (1, 195) = .07, *p* = .79, *η*^2^_p_ < .001 (*M*_American_ = 1.95, *SD*_American_ = .72; *M*_Chinese_ = 1.94, *SD*_Chinese_ = .84). In addition, we tested negative emotional outcomes resulting from deviation from the family-related social clock using a univariate ANOVA on negative emotional outcomes resulting from deviation from the family-related social clock with culture (the U.S. vs. China) as a fixed factor and age, gender, SES, and education as covariates. Similarly, we did not find a significant main effect of culture, *F* (1, 195) = .002, *p* = .97, *η*^2^_p_ < .001 (*M*_American_ = 2.09, *SD*_American_ = .84; *M*_Chinese_ = 2.36, *SD*_Chinese_ = .93).

***Correlations between Filial Piety and the Outcome Measures in the Mediation Analyses in Studies 1a, 1b, and 2***

**Study 1a.** In the U.S., there were negative correlations between filial piety and career-related time window, *r*(341) = -.242, *p* < .001, and between filial piety and family-related time window, *r*(341) = -.287, *p* < .001. In China, there were also negative correlations between filial piety and career-related time window, *r*(295) = -.419, *p* < .001, and between filial piety and family-related time window, *r*(295) = -.414, *p* < .001. Fisher’s *z*-scores for the cultural differences between the correlations for the career-related time window, *z* = −2.51, *p* < .05; for the family-related time window, *z* = −1.82, *p* > .05. Thus, the correlations between filial piety and family-related time windows were not significantly different between the U.S. and China, whereas those between filial piety and career-related time windows were.

**Study 1b.** In the U.S., there were negative correlations between filial piety and career-related time window, *r*(96) = -.283, *p* = .005, and between filial piety and family-related time window, *r*(96) = -.338, *p* < .001. In China, there was a negative correlation between filial piety and career-related time window, *r*(98) = -.234, *p* = .019, but no significant correlation between filial piety and family-related time window, *r*(98) = -.189, *p* = .06. Fisher’s *z*-scores for the cultural differences between the correlations for the career-related time window, *z* = −0.36, *p* > .05; for the family-related time window, *z* = −1.11, *p* > .05. Thus, the correlations between filial piety and career-related or family-related time windows were not significantly different between the U.S. and China.

In the U.S., the correlation between filial piety and anticipated negative emotional outcome of deviation from the career-related social clock was not significant, *r*(96) = .113, *p* = .267, whereas there was a positive correlation between filial piety and anticipated negative emotional outcome of deviation from the family-related social clock, *r*(96) = .360, *p* < .001. In China, there were positive correlations between filial piety and anticipated negative emotional outcome of deviation from the career-related social clock, *r*(98) = .335, *p* < .001, and from the family-related social clock, *r*(98) = .361, *p* < .001. Fisher’s *z*-scores for the cultural differences between the correlations for the career-related time window, *z* = −1.62, *p* > .05; for the family-related time window, *z* = −0.01, *p* > .05. Thus, the correlations between filial piety and anticipated negative emotional outcomes resulting from deviation from career-related or family-related social clocks were not significantly different between the U.S. and China.

**Study 2.** In the U.S., there were negative correlations between filial piety and career-related time window, *r*(90) = -.526, *p* < .001, and between filial piety and family-related time window, *r*(90) = -.324, *p* = .002. In China, there were also negative correlations between filial piety and career-related time window, *r*(107) = -.253, *p* = .008, and between filial piety and family-related time window, *r*(107) = -.269, *p* = .005. Fisher’s *z*-scores for the cultural differences between the correlations for the career-related time window, *z* = −2.27, *p* < .05; for the family-related time window, *z* = −0.42, *p* > .05. Thus, the correlations between filial piety and family-related time windows were not significantly different between the U.S. and China, whereas significant differences were observed in those for career-related time windows.

In sum, Fisher’s z-scores indicated no significant cultural differences in the correlation between filial piety and family-related time windows or well-being outcomes in all studies, while differences were observed in the career-related time windows in Studies 1a and 2.

***Moderation Analyses with Filial Piety***

We ran moderation analyses using the PROCESS macro in SPSS (Hayes, 2018) to examine whether the relationships between filial piety and the outcome measures were moderated by culture with the demographic covariates.

In Study 1a, culture did not moderate the relationship between filial piety and career-related time window (*b* = −2.63 [-6.88, 1.63], *SE* = 2.17, *t*(632) = −1.21, *p* = .225) or between filial piety and family-related time window (*b* = −.84 [-3.99, 2.30], *SE* = 1.60, *t*(632) = −.53, *p* = .598).

In Study 1b, culture did not moderate the relationship between filial piety and career-related time window (*b* = −2.02 [−5.78, 9.81], *SE* = 3.95, *t*(190) = .51, *p* = .610), between filial piety and family-related time window (*b* = 3.48 [−2.10, 9.06], *SE* = 2.83, *t*(190) = 1.23, *p* = .220), between filial piety and anticipated negative emotions of deviation from the upper-bound of the career-related social clock (*b* = .38 [−.13, .90], *SE* = .26, *t*(190) = 1.47, *p* = .144), or between filial piety and anticipated negative emotions of deviation from the upper-bound of the family-related social clock (*b* = .24 [−.26, .75], *SE* = .26, *t*(190) = .94, *p* = .346).

In Study 2, culture moderated the relationship between filial piety and career-related time window (*b* = 8.39 [2.13, 14.65], *SE* = 3.17, *t*(193) = 2.64, *p* = .009) but did not moderate the relationship between filial piety and family-related time window (*b* = 2.44 [−2.75, 7.63], *SE* = 2.63, *t*(193) = .93, *p* = .354). In addition, in Study 2, culture moderated the relationship between filial piety and anticipated negative emotions of deviation from the lower-bound of the career-related social clock (*b* = .51 [.11, .90], *SE* = .20, *t*(193) = 2.55, *p* = .012), but did not moderate the relationship between filial piety and anticipated negative emotions of deviation from the lower-bound of the family-related social clock (*b* = .24 [−.20, .68], *SE* = .22, *t*(193) = 1.08, *p* = .283).

In sum, overall, culture did not consistently moderate the relationship between filial piety and the outcome measures.

***Evaluation of Measurement Invariance of the Filial Piety Scale Across Cultures in Studies 1a, 1b, and 2***

We tested the measurement invariance of the filial piety scale (Fu et al., 2020) across cultures via post-hoc multi-group Confirmatory Factor Analyses (CFA) using the Lavaan package in R (Rosseel, 2012). We assessed the configural invariance, metric invariance, and scalar invariance of the Filial Piety Scale across cultures in each study.

The configural measurement invariance analysis for Study 1a using a Confirmatory Factor Analysis (CFA) model with three factors (Factor 1: caring for parents, Factor 2: familial entirety, and Factor 3: familial aspiration) was conducted across two national cultures (the U.S. and China). Each factor was loaded onto specific filial piety items, as specified in the model. The fit indices for the configural model were as follows, χ²(102) = 486.203, *p* < .001, CFI = .887, RMSEA = .109 (90% CI [.100, .119]), and SRMR = .081. These results indicate that the configural model has an acceptable but not ideal fit, suggesting some potential model misfit. However, the CFI and SRMR values approach the thresholds for acceptable fit, supporting configural invariance across the two national cultures at a preliminary level. The fit indices for the metric model were as follows, χ²(111) = 528.684, *p* < .001, CFI = .877, RMSEA = .109 (90% CI [.100, .119]), and SRMR = .091. Overall, these fit indices suggest that the metric model also has an acceptable but not ideal fit. In addition, the scalar model demonstrated the following fit indices, χ²(120) = 636.629, *p* < .001, CFI = .848, RMSEA = .117 (90% CI [.108, .126]), and SRMR = .102, indicating a slightly suboptimal fit.

We repeated the same analysis with Study 1b. The fitness indices for the configural model, χ²(102) = 216.929, *p* < .001, CFI = .870, RMSEA = .107 (90% CI [.087, .126]), and SRMR = .092, indicates an acceptable but not ideal model fit. The fitness indices for the metric model, χ²(111) = 240.37, *p* < .001, CFI = .854, RMSEA = .109 (90% CI [.090, .127]), and SRMR = .112, and the fitness indices for the scalar model, χ²(120) = 281.02, *p* < .001, CFI = .818, RMSEA = .116 (90% CI [.099, .134]), and SRMR = .122, both suggest a suboptimal, or not ideal, model fit. Lastly, we repeated the same analysis with Study 2. The fit indices for the configural model, χ²(102) = 212.661, *p* < .001, CFI = .880, RMSEA = .104 (90% CI [084, .124], and SRMR = .083, suggest an acceptable but not ideal fit. The fitness indices for the metric model, χ²(111) = 240.15, *p* < .001, CFI = .860, RMSEA = .108 (90% CI [.089, .126]), and SRMR = .107, and the fitness indices for the scalar model, χ²(120) = 263.478, *p* < .001, CFI = .845, RMSEA = .109 (90% CI [.091, .127]), and SRMR = .118, both indicate a suboptimal model fit.

In sum, while the fit indices vary across studies and between levels of invariance (configural, metric, or scalar), the results generally indicate slightly suboptimal model fit.

**References**

Carver, C. S., & White, T. L. (1994). Behavioral inhibition, behavioral activation, and affective responses to impending reward and punishment: the BIS/BAS scales. *Journal of Personality and Social Psychology*, *67*(2), 319- 333. <https://doi.org/10.1037/0022-3514.67.2.319>

Erdfelder, E., Faul, F., & Buchner, A. (1996). Gpower: A general power analysis program. *Behavior Research Methods, Instruments, & Computers*, *28*, 1-11. <https://doi.org/10.3758/BF03203630>

Fu, Y. Y., Xu, Y., & Chui, E. W. T. (2020). Development and Validation of a Filial Piety Scale for Chinese Elders in Contemporary China. *International Journal of Aging and Human Development*, *90*(2), 176-200. <https://doi.org/10.1177/0091415018812399>

Gelfand, M. J., Raver, J. L., Nishii, L., Leslie, L. M., Lun, J., Lim, B. C., Duan, L., Almaliach, A., Ang, S., Arnadottir, J., Aycan, Z., Boehnke, K., Boski, P., Cabecinhas, R., Chan, D., Chhokar, J., D’Amato, A., Ferrer, M., Fischlmayr, I. C., … Yamaguchi, S. (2011). Differences between tight and loose cultures: A 33-nation study. *Science*, *332*(6033), 1100-1104. <https://doi.org/10.1126/science.1197754>

Hayes, A. F. (2018). *Introduction to Mediation, Moderation, and Conditional Process Analysis: A Regression-Based Approach (Methodology in the Social Sciences) (2nd ed.).* New York, NY: The Guilford Press.

Simmons, C. H., Wehner, E. A., Tucker, S. S., & King, C. S. (1988). The cooperative/competitive strategy scale: A measure of motivation to use cooperative or competitive strategies for success. *Journal of Social Psychology*, *128*(2), 199-205. <https://doi.org/10.1080/00224545.1988.9711363>

Zang, L., & Kim, H. S. (in preparation). Culture and the Subjective vs. Intersubjective Social Clock: An Intersubjective Approach to Culture and the Optimal Timing of Life.

Zang, L., & Kim, H. S. (2024, October 23–25). *Culture and judgment of social clock deviation* [Poster presentation]. Association for Psychological Science (APS) Global Psychological Science Summit. Online.

1. Participants read the instructions: People have different understandings regarding what is “on-the-clock.” While being “on-the-clock” could mean you have achieved or are moving towards achieving certain important life milestones within an appropriate age range, you may respond to the following questions based on your personal idea about what is being “on-the-clock”.
2. Some dependent variables in the studies, such as the anticipated emotional outcome variables, meet key statistical assumptions for ANOVA. We report GEEs with robust SEs to maintain consistency.
3. Specifically, we controlled for the lower-bound ages in examining the cultural differences in the time windows, and the main effect of culture remained significant. Specifically, we first examined the cultural differences in the time window for career-related life events. A GEE with robust SEs on the time window for career-related life events with culture (the U.S. vs. China) as a predictor, controlling for demographic covariates and the lower-bound age for career-related life events revealed a significant main effect of culture, Wald χ²(1, *N* = 201) = 51.24, *p* < .001. We then ran the same analysis for the cultural differences in the time window for family-related life events, controlling for demographic covariates and the lower-bound age for family-related life events. Similarly, we found a significant main effect of culture, Wald χ²(1, *N* = 201) = 56.66, *p* < .001. We are unable to control for the upper-bound ages due to high correlations between time windows and upper-bound ages, which would result in problematic multicollinearity (with *r*s > 0.98) if both were included in the same generalized estimating equation (GEE) model.
