# Supplementary material for: Culture and the Social Clock: Cultural Differences in the Optimal Timing of Life
Source: Pers Soc Psychol Bull. 2025 Sep 1;52(10):3144–63. doi: 10.1177/01461672251362514 (PMC13507491; doi:10.1177/01461672251362514)
Supplement: Supplementary file 2 [file sj-docx-2-psp-10.1177_01461672251362514.docx]

**Table 6**

*Descriptive statistics and cultural differences in the time window for each life event, as well as in the lower and upper bound age in Study 2 (controlled for demographic covariates).*

| Specific life event | Culture | Lower bound age  Mean (SD)  Median | Wald χ² (1, *N* = 201) and B for the effect of culture on the lower bound age | Upper bound age  Mean (SD)  Median | Wald χ²(1, *N* = 201) and B for the effect of culture on the upper bound age | Time window Mean (SD)  Median | Wald χ²(1, *N* = 201) and B for the effect of culture on the time window |
| --- | --- | --- | --- | --- | --- | --- | --- |
| Starting college | The U.S. | 17.39 (1.49)  17.00 | Wald χ² = 11.54,  B = -.82 *** | 58.34 (23.77)  57.00 | Wald χ² = 65.08,  B = 29.17 *** | 40.95 (24.06)  40.50 | Wald χ² = 67.74,  B = 29.99 *** |
|  | China | 18.02 (1.45)  18.00 |  | 27.43 (10.06)  23.00 |  | 9.41 (10.01)  6.00 |  |
| Getting a full-time job | The U.S. | 18.95 (2.87)  18.00 | Wald χ² = 56.30,  B = -3.55 *** | 55.93 (18.94)  60.00 | Wald χ² = 54.23,  B = 22.36 *** | 36.99 (19.98)  42.00 | Wald χ² = 65.25,  B = 25.91 *** |
|  | China | 22.57 (2.64)  23.00 |  | 31.67 (9.91)  29.00 |  | 9.10 (10.57)  6.00 |  |
| Getting married (male) | The U.S. | 23.71 (4.24)  24.00 | Wald χ² = 3.23,  B = -1.03 | 67.43 (21.85)  70.00 | Wald χ² = 86.67,  B = 30.49 *** | 43.73 (23.04)  47.50 | Wald χ² = 78.56,  B = 31.51 *** |
|  | China | 24.15 (2.47)  25.00 |  | 34.65 (9.84)  32.00 |  | 10.50 (10.24)  8.00 |  |
| Getting married (female) | The U.S. | 22.82 (3.81)  23.00 | Wald χ² = 1.76,  B = -.71 | 66.92 (22.96)  70.00 | Wald χ² = 85.05,  B = 32.29 *** | 44.11 (23.50)  49.50 | Wald χ² = 81.42,  B = 33.01 *** |
|  | China | 23.03 (2.37)  23.00 |  | 32.50 (9.31)  30.00 |  | 9.48 (9.32)  7.00 |  |
| Having a first child (male) | The U.S. | 25.09 (4.18)  25.00 | Wald χ² = 9.85,  B = -1.79 ** | 46.29 (10.57)  45.00 | Wald χ² = 60.76,  B = 11.58 *** | 21.21 (12.13)  20.00 | Wald χ² = 59.85,  B = 13.36 *** |
|  | China | 26.03 (2.38)  26.00 |  | 34.20 (5.65)  34.00 |  | 8.17 (5.99)  6.00 |  |
| Having a first child (female) | The U.S. | 23.52 (3.68)  23.50 | Wald χ² = 2.61,  B = -.86 | 41.73 (8.95)  40.00 | Wald χ² = 62.29,  B = 9.57 *** | 18.21 (9.47)  16.50 | Wald χ² = 60.05,  B = 10.42 *** |
|  | China | 24.34 (2.70)  25.00 |  | 32.35 (4.71)  32.00 |  | 8.01 (5.16)  6.00 |  |

*** *p* < .001, ** *p* < .01.
